# Effects of microgravity exposure and fructo-oligosaccharide ingestion on the proteome of soleus and extensor digitorum longus muscles in developing mice

**DOI:** 10.1038/s41526-021-00164-6

**Published:** 2021-09-17

**Authors:** Takashi Ohira, Yoko Ino, Yayoi Kimura, Yusuke Nakai, Ayuko Kimura, Yoichi Kurata, Hiroyuki Kagawa, Mitsuo Kimura, Kenji Egashira, Chie Matsuda, Yoshinobu Ohira, Satoshi Furukawa, Hisashi Hirano

**Affiliations:** 1 Research Center for Space and Medical Sciences and Organization for Research Initiatives and Development, Doshisha University, Kyoto, Japan; 2 Department of Physiology and Regenerative Medicine, Kindai University Faculty of Medicine, 377-2 Ohno-Higashi Osaka-Sayama, Osaka, Japan; 3 Advanced Medical Research Center, Yokohama City University, Kanagawa, Japan; 4 Space Biomedical Research Group, Japan Aerospace Exploration Agency, Ibaraki, Japan

**Keywords:** Preventive medicine, Cell biology, Physiology

## Abstract

Short-chain fatty acids produced by the gut bacterial fermentation of non-digestible carbohydrates, e.g., fructo-oligosaccharide (FOS), contribute to the maintenance of skeletal muscle mass and oxidative metabolic capacity. We evaluated the effect of FOS ingestion on protein expression of soleus (Sol) and extensor digitorum longus muscles in mice exposed to microgravity (μ-*g*). Twelve 9-week-old male C57BL/6J mice were raised individually on the International Space Station under μ-*g* or artificial 1-*g* and fed a diet with or without FOS (*n* = 3/group). Regardless of FOS ingestion, the absolute wet weights of both muscles tended to decrease, and the fiber phenotype in Sol muscles shifted toward fast-twitch type following μ-*g* exposure. However, FOS ingestion tended to mitigate the μ-*g*-exposure-related decrease in oxidative metabolism and enhance glutathione redox detoxification in Sol muscles. These results indicate that FOS ingestion mildly suppresses metabolic changes and oxidative stress in antigravity Sol muscles during spaceflight.

## Introduction

Chronic gravitational unloading induces marked muscular atrophy and alters the composition of fiber phenotypes, that is the number of slow-twitch type I fibers decreases, while that of fast-twitch type II or hybrid type I + II fibers increases, especially in antigravity muscles such as the soleus (Sol) and adductor longus^1–7^. Various metabolic changes are also observed in the muscles during gravitational unloading, including decreased oxidative metabolism and increased glycolytic metabolism^8,9^. Additionally, previous studies have demonstrated that the shortening velocity increases, while the peak force and power decrease in Sol muscle fibers of space-flown rats and humans^1–3,6,7^. The functional deterioration of antigravity muscles is gradually recovered in response to gravitational reloading^4,5,10,11^. However, astronauts newly landed on Moon and Mars following their stay in a microgravity (μ-*g*) environment may have trouble carrying out daily activities, since they cannot be directly supported by the local ground staff. This is a serious issue in terms of mission success and crew safety. It is, therefore, crucial to understand the mechanism of skeletal muscle adaptation to gravitational unloading and establish an efficient countermeasure for its prevention during μ-*g* exposure.

To this end, the effect of spaceflight on the transcriptome and proteome profile of murine skeletal muscles has been investigated by comparing the data of spaceflight and ground control (GC) groups^12–16^. However, the spaceflight group was affected not only by μ-*g* exposure, but also by space radiation, high carbon dioxide concentrations, and gravitational reloading associated with atmospheric re-entry and landing on Earth, among other factors. In order to separately evaluate the effects of μ-*g* exposure and other factors, the data obtained from the spaceflight group were compared to those from a hindlimb-suspended group, which was a ground-based simulation group^12,14^. However, the adequacy of this comparison remains debatable, as the stress and behavior of space-flown and hindlimb-suspended rodents differ. Therefore, the Japan Aerospace Exploration Agency (JAXA) developed the Multiple Artificial-gravity Research System (MARS) for raising mice on the International Space Station (ISS) under μ-*g* and artificial 1-*g* (A1-*g*), generated by centrifugation^17,18^. Adopting mice raised under A1-*g* on the ISS as a control group may provide another option to determine specific responses of skeletal muscles to μ-*g* exposure during spaceflight.

Recently, the contribution of gut microbiota to the maintenance of skeletal muscle mass and oxidative metabolic capacity has been demonstrated^19–22^. Bacterially-derived short-chain fatty acids (SCFAs), acetate, butyrate, and propionate, are possible mediators of this effect^19–22^. SCFAs are metabolites produced by the bacterial fermentation of non-digestible carbohydrates in the large intestine^19–22^. Fructo-oligosaccharides (FOS) are one type of non-digestible carbohydrate, and ingestion of 5–6% FOS increases SCFA contents in the cecum of rodents^23–25^. Additionally, the gut-muscle axis attracts the scientific interest as a therapeutic target in the treatment of age-related loss of skeletal muscle mass and function, i.e., sarcopenia^26,27^. Therefore, FOS ingestion could be a countermeasure to prevent the deterioration of antigravity muscle properties in a μ-*g* environment. However, the efficacy of FOS ingestion during spaceflight has not been verified.

In the current study, we isolated Sol and extensor digitorum longus (EDL) muscles from space-flown mice on the second mission using the MARS^17^, and proteome alterations of the muscles in response to μ-*g* exposure were investigated by comparing data obtained from the mice raised under μ-*g* and A1-*g* without FOS ingestion. Furthermore, the efficacy of prebiotic FOS ingestion during spaceflight against the antigravity muscle property deterioration due to μ-*g* exposure was evaluated by comparing the μ-*g*-exposure-related proteome alterations in the muscles of mice fed a diet with and without FOS.

## Results and discussion

### Body and wet weights of skeletal muscles in space-flown mice were affected by decreased food intake during return to Earth

In the current study, a modified AIN-93G diet containing 5% FOS and the energy-equivalent diet containing 5% cellulose instead of FOS were used. Prior to the spaceflight experiment, while mice were raised for 30 days on Earth, the diet containing FOS did not affect the daily food intake or body weight of mice^17^. Additionally, unfavorable somatic symptoms such as indigestion and loose stool were not observed in mice fed a diet containing FOS. Another research team that participated in the second mission using the MARS, analyzed the effects of FOS ingestion on gut microbiota and SCFA contents in the serum and cecum of mice raised on the ISS. They will publish the results in another article (https://www.nasa.gov/mission_pages/station/research/experiments/explorer/Investigation.html?#id=2051).

**Fig. 1:**
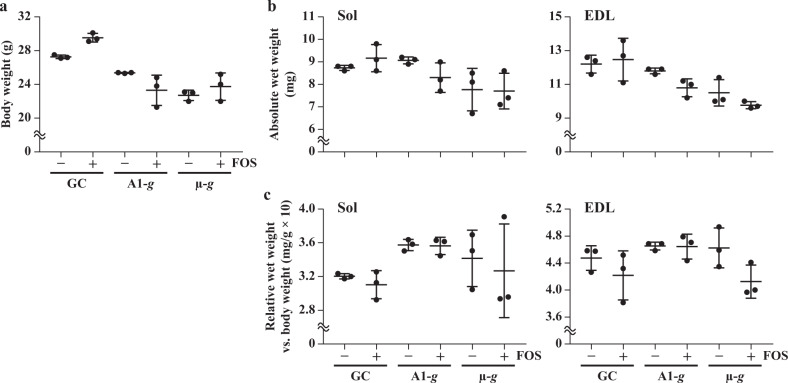
Effects of spaceflight on body weight and wet weight of skeletal muscles of mice, fed a diet with or without fructo-oligosaccharide (FOS). The data are presented as the mean ± standard deviation (*n* = 3). The data pertaining to body weight, absolute wet weight of soleus (Sol) and extensor digitorum longus (EDL) muscles, and the wet weight of Sol and EDL muscles relative to body weight are depicted in panels (**a**−**c**), respectively. Ground control (GC): group of mice raised in the ground model of the Multiple Artificial-gravity Research System habitat and transportation cage units; artificial 1-*g* (A1-*g*): group of mice raised under artificial 1-*g* on the International Space Station (ISS); microgravity (μ-*g*): group of mice raised under μ-*g* on the ISS; and FOS (+) or (−): groups of mice fed a diet with or without FOS, respectively.

All 12 mice, which were individually raised on the ISS, returned alive to Earth and survived until the tissue sampling. Mice in the GC group were individually raised in the ground model of the MARS habitat and transportation cage units under the same conditions as the A1-*g* and μ-*g* groups. Large standard deviations (SDs) were observed in the body weights of mice fed a diet containing 5% FOS in both A1-*g* and μ-*g* groups (Fig. 1a). Microgravity exposure and FOS ingestion did not significantly influence body weight (*n* = 3/group). However, statistical analyses of the integrated data of mice fed a diet with or without FOS (*n* = 6/group) revealed that the body weights of mice in the A1-*g* (*p* = 0.0446) and μ-*g* (*p* = 0.0019) groups were significantly lower than those of mice in the GC group (Supplementary Fig. 1a). These results could be attributed to the decreased food intake of mice in the A1-*g* and μ-*g* groups, in response to the altered gravitational force^28–30^.

On the mission by JAXA, space-flown mice were relocated from habitat to transportation cage units of the MARS before returning to Earth^17,18^. The space-flown mice in this study were dissected within 36.5 h after splashdown of the MARS transportation cage unit in the Pacific Ocean^17^. Mice in the GC group were also relocated to the transportation cage unit and raised for the same period as the A1-*g* and µ-*g* groups until the dissection. The food intake of the GC, A1-*g*, and µ-*g* groups in the transportation cage was 7.1 ± 1.1, 4.4 ± 1.3, and 1.9 ± 2.5 g (mean ± SD), respectively. Although these values were estimated based on the difference between the mean weight of the cylindrical food bars equipped in the MARS transportation cages^18^ before returning to Earth and the weight of the remaining food bar in each cage, the food intake of the A1-*g* and µ-*g* groups was 38.2 and 72.6% lower, respectively, than that of the GC group. The decrease in food intake would be attributed to the acute response of the vestibular system to hypergravity and terrestrial gravity exposure after leaving the ISS and returning to Earth^31,32^.

The absolute wet weight of both Sol and EDL muscles tended to be decreased by µ-*g* exposure (Fig. 1b). Raising mice under A1-*g* on the ISS tended to be effective in mitigating the decrease in the weight of antigravity Sol muscles but FOS ingestion was not. Furthermore, FOS ingestion tended to decrease the absolute wet weight of Sol and EDL muscles in mice raised under A1-*g* on the ISS. However, no significant differences were observed between any of the groups (*n* = 3/group) with respect to absolute wet weight (Fig. 1b) and the wet weight of Sol and EDL muscles relative to body weight (Fig. 1c). Meanwhile, statistical analyses using the integrated data obtained from mice fed a diet with or without FOS (*n* = 6/group) revealed that the absolute wet weight of Sol (−13.6%, *p* = 0.0299) and EDL (−17.8%, *p* = 0.0032) muscles in the μ-*g* group was significantly lower than that in the GC group (Supplementary Fig. 1b). The mean absolute wet weight of Sol muscles in the μ-*g* group was 10.9% lower than that of the A1-*g* group; however, this difference was not statistically significant (*p* = 0.0635). No significant differences were observed between any of the groups with respect to the relative wet weight of Sol and EDL muscles (Supplementary Fig. 1c).

Similar changes were observed in the wet weight of Sol and EDL muscles in space-flown mice on the Bion-M1 Space Mission, in which mouse skeletal muscles were isolated approximately 13−16 h after landing^16^. The reduction in a wet weight of Sol muscles from mice in the μ-*g* group (Supplementary Fig. 1b) could be attributed to suppressed growth and high susceptibility to gravitational unloading^1–7^, and indicated no evidence that FOS ingestion suppressed the atrophy of Sol muscles in mice exposed to μ-*g*. The low absolute wet weight of EDL muscles in mice exposed to μ-*g* (Supplementary Fig. 1b) could be attributed to suppressed growth and decreased food intake in response to exposure to hypergravity and terrestrial gravity after leaving the ISS and returning to Earth. Previous studies have reported a decrease in the food intake of mice acutely exposed to 1.4-*g*^28^ and 2-*g*^29^. Additionally, autophagy is known to be prominently induced in fast-twitch EDL, tibialis anterior, and plantaris muscles compared to slow-twitch Sol muscles of mice and rats starved for 24 h^33–35^.

These results indicated that comparisons between the GC and μ-*g* groups would highlight not only the effect of μ-*g* exposure, but also the effect of decreased food intake on skeletal muscles. On the other hand, mice raised under A1-*g* on the ISS would serve as a more suitable control group for determining the specific responses of the skeletal muscle proteome to μ-*g* exposure during spaceflight.

### Protein abundance profile in Sol and EDL muscles was altered in response to both µ-*g* exposure and FOS ingestion

The abundance of 1,253 proteins in Sol and EDL muscles from mice in the μ-*g* and A1-*g* groups, fed a diet with or without FOS (*n* = 3/group), were individually identified by proteomic analysis using liquid chromatography coupled with mass spectrometry (LC-MS/MS) and label-free quantitation. Proteins were extracted from each muscle by a two-step solubilization method^36^ to increase the coverage of proteins identified by LC-MS/MS analysis. The cellular components of extracted proteins in supernatant-1 (SUP-1) and supernatant-2 (SUP-2) were similar to that observed in our previous study^36^ (Supplementary Table 1).

**Fig. 2:**
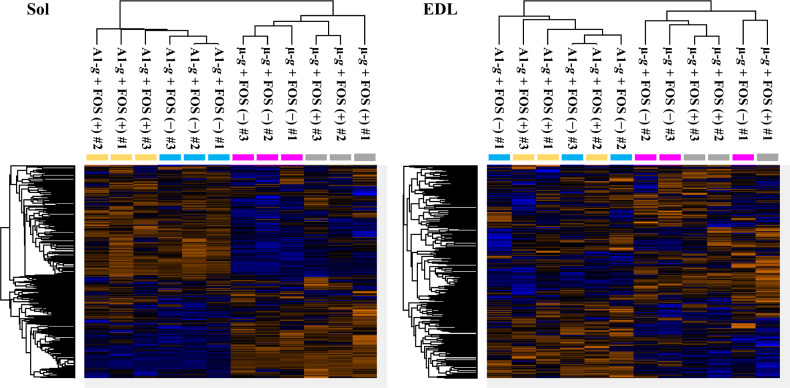
Classification of soleus (Sol) and extensor digitorum longus (EDL) muscle samples according to the abundance of 1,253 proteins. The abundance of each protein is depicted in a gradient from blue (low) to orange (high). Four groups, namely, the artificial 1-*g* (A1-*g*) and fructo-oligosaccharide (FOS) (+), A1-*g* and FOS (−), microgravity (µ-*g*) and FOS (+), and µ-*g* and FOS (−) groups, are indicated in yellow, cyan, magenta, and gray, respectively.

Heatmaps and clustering of analyzed samples according to the abundance of 1,253 proteins demonstrated that both Sol and EDL muscles responded to μ-*g* exposure (Fig. 2). Additionally, Sol muscles from mice exposed to μ-*g* and A1-*g* were affected by FOS ingestion.

The protein abundance profiles in Sol and EDL muscles of the groups exposed to µ-*g* with and without FOS ingestion and the group exposed to A1-*g* with FOS ingestion were individually compared with that of the group exposed to A1-*g* without FOS ingestion to evaluate the response of muscle proteins to μ-*g* or A1-*g* exposure and/or FOS ingestion (Supplementary Fig. 2). Particularly responsive proteins in both muscles were selected based on *p* values (< 0.01) and fold changes (> 2.0) and are listed in Supplementary Table 2. None of the proteins in the group exposed to A1-*g* with FOS ingestion meet these criteria. The response of several proteins, especially in Sol muscles, to μ-*g* exposure was mitigated by FOS ingestion. However, the response of other proteins was enhanced by the combined effect of μ-*g* exposure and FOS ingestion.

In Sol muscles from mice exposed to μ-*g*, changes in metabolic processes associated with the shift of fiber phenotype from slow- to fast-twitch type were clearly observed (Supplementary Tables 3 and 4). Proteins involved in the glycolytic process were significantly increased, while those involved in fatty acid beta-oxidation, tricarboxylic acid cycle, and mitochondrial electron transport were significantly decreased (Table 1). Additionally, a significant increase in proteins involved in the glutathione metabolic process, glutathione-mediated detoxification, and glutathione redox reactions was observed, especially in Sol muscles of mice exposed to μ-*g* (Tables 2 and 3 and Supplementary Tables 3 and 4). Previous studies reported similar changes in the proteome of Sol muscles from space-flown mice^16^ and humans^37^. Ingestion of FOS tended to mitigate the μ-*g*-exposure-related changes in metabolic processes in Sol muscles and enhance the activation of glutathione redox reactions and glutathione-mediated detoxification (Tables 1 and 2). Biological interpretations using Ingenuity Pathway Analysis of the significant alteration of protein abundance profiles in Sol muscles (*p* < 0.01) in response to μ-*g* exposure and/or FOS ingestion were also identical (Table 4).

**Table 1:** Responses of soleus (Sol) muscle proteins involved in metabolic processes to microgravity (µ-*g*) or artificial 1-*g* (A1-*g*) exposure and/or fructo-oligosaccharide (FOS) ingestion.

Gene symbol (accession)	µ-*g* & FOS (−)/A1-*g* & FOS (−)	µ-*g* & FOS (+)/A1-*g* & FOS (−)	A1-*g* & FOS (+)/A1-*g* & FOS (−)
Glycolytic process			
Aldoa (P05064)	* FC = 1.78, *P* = 1.5E−4	* FC = 1.53, *P* = 4.8E−3	FC = 0.96, *P* = 5.1E−1
Gpi1 (P06745)	* FC = 1.76, *P* = 2.8E−3	* FC = 1.80, *P* = 3.8E−3	FC = 1.09, *P* = 3.5E−1
Gapdh (P16858)	* FC = 1.53, *P* = 6.8E−3	FC = 1.53, *P* = 3.7E−2	FC = 1.04, *P* = 8.2E−1
Pfkm (P47857)	* FC = 1.21, *P* = 3.8E−3	FC = 1.02, *P* = 6.8E−1	FC = 0.90, *P* = 6.4E−2
Pgk1 (P09411)	* FC = 1.62, *P* = 8.8E−4	* FC = 1.69, *P* = 7.9E−3	* FC = 1.18, *P* = 5.8E−3
Pgk2 (P09041)	* FC = 3.04, *P* = 3.7E−3	FC = 2.97, *P* = 1.1E−2	FC = 1.48, *P* = 5.9E−2
Pkm (P52480)	* FC = 1.46, *P* = 2.5E−3	FC = 1.37, *P* = 1.4E−2	FC = 1.05, *P* = 4.2E−1
Fatty acid beta-oxidation			
Acat1 (Q8QZT1)	* FC = 0.68, *P* = 2.9E−4	* FC = 0.75, *P* = 2.2E−3	FC = 0.93, *P* = 8.3E−2
Acaa1b (Q8VCH0)	* FC = 0.59, *P* = 8.2E−3	FC = 0.76, *P* = 1.3E−1	FC = 0.85, *P* = 2.3E−1
Acaa2 (Q8BWT1)	* FC = 0.63, *P* = 1.8E−3	* FC = 0.71, *P* = 1.0E−3	FC = 0.95, *P* = 2.7E−1
Acad10 (Q8K370)	FC = 0.74, *P* = 1.1E−2	* FC = 0.80, *P* = 1.6E−3	FC = 0.94, *P* = 3.6E−1
Acadl (P51174)	FC = 0.78, *P* = 1.1E−2	* FC = 0.85, *P* = 9.5E−4	FC = 0.94, *P* = 1.6E−1
Acadm (P45952)	* FC = 0.53, *P* = 1.5E−3	* FC = 0.61, *P* = 2.8E−4	FC = 0.94, *P* = 2.3E−1
Acadsb (Q9DBL1)	* FC = 0.70, *P* = 5.9E−3	FC = 0.83, *P* = 6.4E−2	FC = 0.90, *P* = 2.8E−1
Acadvl (P50544)	* FC = 0.69, *P* = 3.4E−4	* FC = 0.78, *P* = 4.1E−3	FC = 0.93, *P* = 1.7E−1
Acox1 (Q9R0H0)	* FC = 0.15, *P* = 8.0E−4	* FC = 0.20, *P* = 1.9E−3	FC = 0.88, *P* = 5.2E−1
Cpt1b (Q924X2)	* FC = 0.71, *P* = 3.0E−3	FC = 0.74, *P* = 1.6E−2	FC = 1.02, *P* = 7.4E−1
Decr1 (Q9CQ62)	* FC = 0.68, *P* = 1.3E−3	* FC = 0.77, *P* = 6.0E−3	FC = 0.98, *P* = 7.3E−1
Etfa (Q99LC5)	* FC = 0.72, *P* = 3.0E−3	FC = 0.88, *P* = 5.1E−2	FC = 0.95, *P* = 3.3E−1
Etfdh (Q921G7)	* FC = 0.70, *P* = 2.2E−3	FC = 0.80, *P* = 1.1E−2	FC = 0.96, *P* = 5.8E−1
Ech1 (O35459)	* FC = 0.38, *P* = 6.1E−4	* FC = 0.46, *P* = 1.1E−3	FC = 0.92, *P* = 3.7E−1
Eci1 (P42125)	* FC = 0.64, *P* = 1.8E−3	* FC = 0.72, *P* = 1.1E−3	FC = 0.93, *P* = 3.4E−1
Eci2 (Q9WUR2)	* FC = 0.71, *P* = 1.5E−3	FC = 0.81, *P* = 1.8E−2	FC = 0.99, *P* = 7.7E−1
Hadh (Q61425)	* FC = 0.63, *P* = 3.4E−3	* FC = 0.74, *P* = 6.4E−3	FC = 0.88, *P* = 1.3E−1
Hadha (Q8BMS1)	* FC = 0.62, *P* = 1.6E−4	FC = 0.74, *P* = 1.5E−2	FC = 0.97, *P* = 6.2E−1
Hadhb (Q99JY0)	* FC = 0.65, *P* = 9.9E−5	FC = 0.78, *P* = 1.3E−2	FC = 0.95, *P* = 4.5E−1
Hibch (Q8QZS1)	* FC = 0.74, *P* = 2.2E−3	FC = 0.87, *P* = 2.2E−2	FC = 0.94, *P* = 5.4E−2
Tricarboxylic acid cycle			
Cs (Q9CZU6)	* FC = 0.79, *P* = 6.5E−4	* FC = 0.87, *P* = 2.7E−3	FC = 0.93, *P* = 5.4E−2
Dlat (Q8BMF4)	* FC = 0.74, *P* = 5.0E−3	FC = 0.83, *P* = 1.1E−2	FC = 0.92, *P* = 2.0E−1
Dlst (Q9D2G2)	* FC = 0.75, *P* = 3.8E−3	* FC = 0.81, *P* = 7.4E−3	FC = 0.90, *P* = 8.6E−2
Fh1 (P97807)	* FC = 0.81, *P* = 5.9E−3	FC = 1.00, *P* = 1.0	FC = 0.88, *P* = 4.8E−2
Idh2 (P54071)	* FC = 0.57, *P* = 4.4E−4	* FC = 0.70, *P* = 3.9E−3	FC = 1.04, *P* = 6.6E−1
Idh3g (P70404)	* FC = 0.76, *P* = 7.1E−3	FC = 0.82, *P* = 1.7E−2	FC = 0.85, *P* = 2.1E−2
Mdh2 (P08249)	* FC = 0.82, *P* = 4.1E−3	FC = 0.91, *P* = 1.6E−1	FC = 0.91, *P* = 6.4E−2
Ogdh (Q60597)	* FC = 0.74, *P* = 7.8E−4	* FC = 0.83, *P* = 3.7E−3	FC = 0.88, *P* = 1.1E−1
Pdhb (Q9D051)	* FC = 0.82, *P* = 5.2E−3	FC = 0.91, *P* = 5.5E−2	FC = 0.92, *P* = 2.3E−1
Sdha (Q8K2B3)	* FC = 0.82, *P* = 7.4E−3	FC = 0.89, *P* = 7.5E−2	FC = 0.91, *P* = 1.8E−1
Mitochondrial electron transport			
Cox2 (P00405)	* FC = 0.69, *P* = 6.9E−3	FC = 0.78, *P* = 3.0E−2	FC = 0.84, *P* = 1.0E−1
Cox4i1 (P19783)	* FC = 0.78, *P* = 1.6E−3	* FC = 0.80, *P* = 4.0E−3	FC = 0.91, *P* = 1.4E−1
Cox5a (P12787)	* FC = 0.76, *P* = 7.7E−3	FC = 0.87, *P* = 1.6E−1	FC = 1.03, *P* = 7.8E−1
(Cox5b (P19536)	* FC = 0.82, *P* = 8.5E−3	FC = 0.90, *P* = 3.8E−1	FC = 0.96, *P* = 6.2E−1
Cox7c (P17665)	* FC = 0.68, *P* = 4.7E−3	FC = 0.81, *P* = 4.2E−2	FC = 0.92, *P* = 4.7E−1
Cycs (P62897)	* FC = 0.66, *P* = 1.6E−3	* FC = 0.70, *P* = 8.6E−3	FC = 0.95, *P* = 4.0E−1
Pmpcb (Q9CXT8)	* FC = 0.62, *P* = 2.9E−3	FC = 0.86, *P* = 4.8E−1	FC = 0.81, *P* = 1.0E−1
Uqcrc2 (Q9DB77)	* FC = 0.83, *P* = 2.5E−4	FC = 0.91, *P* = 1.7E−1	FC = 0.89, *P* = 8.6E−2

The protein abundance profiles of the groups exposed to µ-*g* with and without FOS ingestion and the group exposed to A1-*g* with FOS ingestion were individually compared with that of the group exposed to A1-*g* without FOS ingestion. The results for the proteins, which were categorized as the related factors to each metabolic process by DAVID Bioinformatics Resources 6.8 and significantly (*p* < 0.01) affected by µ-*g* exposure and/or FOS ingestion, in Sol muscles are indicated. The fold change (FC) and *p* value (*P*) of each protein are depicted. *: the significant high- or low-abundance proteins.

**Table 2:** Responses of soleus (Sol) muscle proteins involved in glutathione-mediated detoxification and glutathione redox reactions to microgravity (µ-*g*) or artificial 1-*g* (A1-*g*) exposure and/or fructo-oligosaccharide (FOS) ingestion.

Gene symbol (accession)	µ-*g* & FOS (−)/A1-*g* & FOS (−)	µ-*g* & FOS (+)/A1-*g* & FOS (−)	A1-*g* & FOS (+)/A1-*g* & FOS (−)
Gpx1 (P11352)	FC = 1.26, *P* = 5.0E−2	* FC = 1.55, *P* = 6.1E−3	FC = 1.03, *P* = 6.4E−1
Gpx3 (P46412)	* FC = 2.89, *P* = 9.4E−4	* FC = 3.55, *P* = 1.2E−4	FC = 1.08, *P* = 1.7E−1
Gsr (P47791)	* FC = 1.43, *P* = 4.1E−3	* FC = 1.38, *P* = 5.7E−3	FC = 1.04, *P* = 8.9E−1
Gstk1 (Q9DCM2)	* FC = 0.77, *P* = 1.5E−4	FC = 1.02, *P* = 8.0E−1	FC = 0.99, *P* = 8.3E−1
Gstm1 (P10649)	* FC = 1.91, *P* = 1.5E−4	* FC = 2.02, *P* = 3.4E−4	FC = 1.00, *P* = 9.1E−1
Gstm2 (P15626)	* FC = 1.64, *P* = 1.7E−3	* FC = 1.83, *P* = 6.0E−4	FC = 0.96, *P* = 6.0E−1
Gstm5 (P48774)	FC = 1.20, *P* = 1.4E−2	* FC = 1.48, *P* = 6.2E−4	FC = 0.99, *P* = 8.1E−1
Gsto1 (O09131)	FC = 0.87, *P* = 1.4E−1	FC = 0.97, *P* = 6.3E−1	* FC = 1.26, *P* = 6.3E−3
Gstp1 (P19157)	* FC = 1.82, *P* = 9.9E−4	* FC = 2.50, *P* = 1.4E−3	FC = 1.39, *P* = 4.9E−2

The protein abundance profiles of the groups exposed to µ-*g* with and without FOS ingestion and the group exposed to A1-*g* with FOS ingestion were individually compared with that of the group exposed to A1-*g* without FOS ingestion. The results for the proteins, which were categorized as the related factors to glutathione metabolic process, glutathione-mediated detoxification and/or glutathione redox reactions by DAVID Bioinformatics Resources 6.8 and Ingenuity Pathway Analysis and significantly (*p* < 0.01) affected by µ-*g* exposure and/or FOS ingestion, in Sol muscles are indicated. The fold change (FC) and *p* value (*P*) of each protein are depicted. *: the significant high- or low-abundance proteins.

**Table 3:** Responses of extensor digitorum longus (EDL) muscle proteins involved in glutathione-mediated detoxification and glutathione redox reactions to microgravity (µ-*g*) or artificial 1-*g* (A1-*g*) exposure and/or fructo-oligosaccharide (FOS) ingestion.

Gene symbol (accession)	µ-*g* & FOS (−)/A1-*g* & FOS (−)	µ-*g* & FOS (+)/A1-*g* & FOS (−)	A1-*g* & FOS (+)/A1-*g* & FOS (−)
Gsr (P47791)	* FC = 1.21, *P* = 2.6E−3	FC = 1.30, *P* = 1.1E−2	FC = 0.92, *P* = 2.0E−2
Gsto1 (O09131)	* FC = 1.30, *P* = 2.2E−3	* FC = 1.41, *P* = 1.6E−4	FC = 1.00, *P* = 9.8E−1

The protein abundance profiles of the groups exposed to µ-*g* with and without FOS ingestion and the group exposed to A1-*g* with FOS ingestion were individually compared with that of the group exposed to A1-*g* without FOS ingestion. The results for the proteins, which were categorized as the related factors to glutathione metabolic process, glutathione-mediated detoxification and/or glutathione redox reactions by DAVID Bioinformatics Resources 6.8 and Ingenuity Pathway Analysis and significantly (*p* < 0.01) affected by µ-*g* exposure and/or FOS ingestion, in EDL muscles are indicated. The fold change (FC) and *p* value (*P*) of each protein are depicted. *: the significant high- or low-abundance proteins.

**Table 4:** Biological implications using Ingenuity Pathway Analysis for the altered protein abundance profiles of soleus (Sol) muscles by microgravity (µ-*g*) exposure and/or fructo-oligosaccharide (FOS) ingestion.

Canonical pathways	µ-*g* & FOS (−)/A1-*g* & FOS (−)	µ-*g* & FOS (+)/A1-*g* & FOS (−)
Glycolysis I	2.65	2
Oxidative phosphorylation	−4.69	−2.45
Fatty acid β-oxidation I	−3.16	−2
TCA cycle II (Eukaryotic)	−2.65	N.D.
Glutathione-mediated detoxification	1	2
Glutathione redox reactions I	1.34	2.24

The protein abundance profiles in Sol muscles of the groups exposed to µ-*g* with and without FOS ingestion were individually compared with that of the group exposed to artificial 1-*g* (A1-*g*) without FOS ingestion. The biological effect of protein abundance profiles which significantly (*p* < 0.01) altered by µ-*g* exposure and/or FOS ingestion on canonical pathways related to metabolic process, glutathione-mediated detoxification, and glutathione redox reactions were interpreted with Ingenuity Pathway Analysis. *Z* scores, which were calculated to presume activation or inactivation of each index, are depicted. Notable biological effects on extensor digitorum longus muscles were not indicated by Ingenuity Pathway Analysis. *N.D* not determined.

These effects would be attributed to the increased bacterially-derived SCFAs, acetate, butyrate, and propionate, in the gut of mice fed a diet containing 5% FOS, as has been reported previously^23–25^. The SCFAs produced in the gut are transported into the circulatory system in vivo, and the SCFA receptors, free fatty acid receptor (Ffar) 2 and Ffar3, are present in skeletal muscle tissue^19,38,39^. There is growing evidence that SCFAs activate AMP-activated protein kinase in skeletal muscles of mice and enhance the oxidative metabolic capacity^19,20,40–42^. Walsh et al.^42^ reported that butyrate ingestion enhanced mitochondrial biogenesis and reduced oxidative stress in skeletal muscles of C57BL/6 female mice aged 26 months. However, in the current study, the abundance of proteins that composed sarcomere and affected muscle contractile properties was significantly altered in association with the shift of fiber phenotype from slow- to fast-twitch type, even in Sol muscles of mice exposed to μ-*g* with FOS ingestion (Table 5). These results indicated that the effect of FOS ingestion was not sufficient to suppress the phenotype shift toward fast-twitch type in Sol muscles of mice exposed to μ-*g*.

**Table 5:** Responses of soleus (Sol) muscle proteins composing sarcomere and affecting muscle contractile properties to microgravity (µ-*g*) or artificial 1-*g* (A1-*g*) exposure and/or fructo-oligosaccharide (FOS) ingestion.

Gene symbol (accession)	µ-*g* & FOS (−)/A1-*g* & FOS (−)	µ-*g* & FOS (+)/A1-*g* & FOS (−)	A1-*g* & FOS (+)/A1-*g* & FOS (−)
Predominant proteins in slow-twitch muscle			
Actn2 (Q9JI91)	* FC = 0.82, *P* = 1.1E−3	* FC = 0.88, *P* = 9.6E−4	FC = 1.09, *P* = 7.5E−2
Atp2a2 (O55143)	* FC = 0.71, *P* = 8.0E−3	* FC = 0.69, *P* = 2.0E−5	* FC = 1.27, *P* = 2.7E−3
Myl6b (Q8CI43)	* FC = 0.43, *P* = 6.3E−3	* FC = 0.47, *P* = 3.6E−3	FC = 1.44, *P* = 9.7E−2
Myl2 (P51667)	* FC = 0.61, *P* = 3.2E−3	FC = 0.70, *P* = 1.3E−2	FC = 1.21, *P* = 8.4E−2
Myh7 (Q91Z83)	FC = 0.77, *P* = 3.5E−2	* FC = 0.83, *P* = 4.4E−3	* FC = 1.38, *P* = 3.7E−4
Myh7b (A2AQP0)	* FC = 0.36, *P* = 2.3E−3	* FC = 0.44, *P* = 2.5E−3	FC = 1.05, *P* = 7.5E−1
Myoz2 (Q9JJW5)	* FC = 0.53, *P* = 7.5E−5	* FC = 0.64, *P* = 1.9E−3	FC = 1.09, *P* = 1.0E−1
Tpm3 (P21107)	* FC = 0.68, *P* = 8.8E−3	FC = 0.71, *P* = 1.7E−2	FC = 1.14, *P* = 2.1E−1
Predominant proteins in fast-twitch muscle			
Actn3 (O88990)	* FC = 2.66, *P* = 3.7E−5	* FC = 1.82, *P* = 1.4E−3	FC = 1.08, *P* = 5.2E−1
Atp2a1 (Q8R429)	* FC = 1.60, *P* = 2.4E−4	* FC = 1.44, *P* = 9.1E−4	FC = 1.00, *P* = 8.5E−1
Casq1 (O09165)	FC = 1.27, *P* = 8.9E−2	* FC = 1.35, *P* = 3.0E−3	FC = 1.04, *P* = 5.7E−1
Mylk2 (Q8VCR8)	* FC = 1.51, *P* = 6.9E−3	* FC = 1.48, *P* = 4.3E−3	FC = 0.94, *P* = 2.9E−1
Myh4 (Q5SX39)	* FC = 4.78, *P* = 7.1E−4	* FC = 3.95, *P* = 2.4E−3	FC = 1.08, *P* = 8.6E−1
Mybpc2 (Q5XKE0)	* FC = 1.85, *P* = 2.6E−4	FC = 1.36, *P* = 3.7E−2	FC = 1.10, *P* = 3.4E−1

The protein abundance profiles of the groups exposed to µ-*g* with and without FOS ingestion and the group exposed to A1-*g* with FOS ingestion were individually compared with that of the group exposed to A1-*g* without FOS ingestion. The results for the proteins, which were categorized as the components of sarcomere and/or related factors to skeletal muscle contraction by DAVID Bioinformatics Resources 6.8 and significantly (*p* < 0.01) affected by µ-*g* exposure and/or FOS ingestion, in Sol muscles are indicated. The fold change (FC) and *p* value (*P*) of each protein are depicted. *: the significant high- or low-abundance proteins.

In contrast to Sol muscles, there was a trend of increased oxidative metabolism and decreased glycolytic metabolism in EDL muscles of mice exposed to μ-*g* (Table 6 and Supplementary Tables 5 and 6). These changes were more prominent in mice exposed to μ-*g* with FOS ingestion. This result would be attributed to the effect of gut-derived SCFAs promoting oxidative metabolism in skeletal muscles^19,20,40–42^. On the other hand, in addition to a trend of slight activation of the mitochondrial tricarboxylic acid cycle (Table 6 and Supplementary Tables 5 and 6), a trend of the shift of fiber phenotype from fast- to slow-twitch type (Table 7) in EDL muscles of mice exposed to μ-*g* strongly suggested that EDL muscles were chronically stretched, and their neuro-muscular activities were altered from phasic to chronic pattern in a μ-*g* environment. The tibialis anterior muscle, which is located at the same tibial side as the EDL, is passively stretched in response to plantar flexion of the ankle joint during gravitational unloading^43,44^. In contrast to calf muscles such as the Sol and gastrocnemius, the electromyographic activity of the tibialis anterior muscle increases even during hindlimb suspension and μ-*g* exposure^45,46^. Similarly, the electromyographic activity of mouse EDL muscles was probably increased during μ-*g* exposure in the current study, because the digits in the hindlimbs of mice floating in the MARS habitat cage had been opened and extended^18^.

**Table 6:** Responses of extensor digitorum longus (EDL) muscle proteins involved in metabolic processes to microgravity (µ-*g*) or artificial 1-*g* (A1-*g*) exposure and/or fructo-oligosaccharide (FOS) ingestion.

Gene symbol (accession)	µ-*g* & FOS (−)/A1-*g* & FOS (−)	µ-*g* & FOS (+)/A1-*g* & FOS (−)	A1-*g* & FOS (+)/A1-*g* & FOS (−)
Glycolytic process			
Aldoa (P05064)	FC = 0.92, *P* = 6.1E−2	* FC = 0.88, *P* = 7.7E−3	FC = 1.02, *P* = 1.0E−1
Bpgm (P15327)	FC = 0.84, *P* = 6.6E−2	* FC = 0.84, *P* = 6.9E−4	FC = 1.05, *P* = 4.8E−1
Pfkm (P47857)	FC = 0.78, *P* = 2.4E−2	* FC = 0.55, *P* = 3.0E−3	FC = 0.92, *P* = 4.1E−1
Fatty acid beta-oxidation			
Acox1 (Q9R0H0)	FC = 2.26, *P* = 2.7E−2	* FC = 3.70, *P* = 8.1E−3	FC = 1.71, *P* = 3.8E−2
Etfa (Q99LC5)	FC = 1.13, *P* = 1.1E−2	* FC = 1.17, *P* = 2.4E−3	FC = 1.00, *P* = 9.6E−1
Etfb (Q9DCW4)	FC = 1.15, *P* = 1.5E−2	* FC = 1.19, *P* = 3.5E−3	FC = 1.01, *P* = 6.4E−1
Tricarboxylic acid cycle			
Aco1 (P28271)	* FC = 1.21, *P* = 5.9E−3	FC = 1.21, *P* = 8.0E−2	FC = 0.96, *P* = 3.7E−1
Idh2 (P54071)	* FC = 1.43, *P* = 8.5E−4	* FC = 1.45, *P* = 3.2E−4	FC = 0.98, *P* = 6.3E−1
Mdh1 (P14152)	FC = 1.05, *P* = 1.4E−1	* FC = 1.09, *P* = 6.2E−3	FC = 0.99, *P* = 8.0E−1
Ogdh (Q60597)	FC = 1.11, *P* = 3.1E−2	* FC = 1.09, *P* = 7.3E−4	FC = 1.01, *P* = 5.6E−1
Pdha1 (P35486)	* FC = 1.15, *P* = 5.2E−4	FC = 1.16, *P* = 1.8E−2	FC = 1.05, *P* = 3.6E−2
Suclg2 (Q9Z2I8)	FC = 1.36, *P* = 2.4E−2	* FC = 1.63, *P* = 5.1E−3	FC = 0.93, *P* = 5.5E−1

The protein abundance profiles of the groups exposed to µ-*g* with and without FOS ingestion and the group exposed to A1-*g* with FOS ingestion were individually compared with that of the group exposed to A1-*g* without FOS ingestion. The results for the proteins, which were categorized as the related factors to each metabolic process by DAVID Bioinformatics Resources 6.8 and significantly (*p* < 0.01) affected by µ-*g* exposure and/or FOS ingestion, in EDL muscles, are indicated. The fold change (FC) and *p* value (*P*) of each protein are depicted. *: the significant high- or low-abundance proteins.

**Table 7:** Responses of extensor digitorum longus (EDL) muscle proteins composing sarcomere and affecting muscle contractile properties to microgravity (µ-*g*) or artificial 1-*g* (A1-*g*) exposure and/or fructo-oligosaccharide (FOS) ingestion.

Gene symbol (accession)	µ-*g* & FOS (−)/A1-*g* & FOS (−)	µ-*g* & FOS (+)/A1-*g* & FOS (−)	A1-*g* & FOS (+)/A1-*g* & FOS (−)
Predominant proteins in slow-twitch muscle			
Actn2 (Q9JI91)	FC = 1.17, *P* = 3.5E−2	* FC = 1.28, *P* = 6.2E−3	FC = 1.04, *P* = 4.8E−1
Myh7b (A2AQP0)	FC = 1.48, *P* = 3.5E−2	* FC = 1.94, *P* = 5.1E−3	FC = 0.96, *P* = 8.3E−1
Myoz2 (Q9JJW5)	* FC = 1.51, *P* = 6.3E−4	* FC = 1.71, *P* = 1.9E−4	FC = 0.95, *P* = 5.0E−1
Predominant proteins in fast-twitch muscle			
Mybpc2 (Q5XKE0)	FC = 0.86, *P* = 1.1E−1	* FC = 0.73, *P* = 4.3E−3	FC = 0.95, *P* = 5.0E−1

The protein abundance profiles of the groups exposed to µ-*g* with and without FOS ingestion and the group exposed to A1-*g* with FOS ingestion were individually compared with that of the group exposed to A1-*g* without FOS ingestion. The results for the proteins, which were categorized as the components of sarcomere and/or related factors to skeletal muscle contraction by DAVID Bioinformatics Resources 6.8 and significantly (*p* < 0.01) affected by µ-*g* exposure and/or FOS ingestion, in EDL muscles are indicated. The fold change (FC) and *p* value (*P*) of each protein are depicted. *: the significant high- or low-abundance proteins.

We also observed that the abundance of blood microparticle and extracellular exosome proteins in Sol and EDL muscles was significantly affected by μ-*g* exposure, regardless of FOS ingestion (Supplementary Tables 3−6). However, the abundance of several proteins in these categories differed between mice with and without FOS ingestion (Supplementary Table 7). This effect might be partly attributed to the changes in metabolic properties of these muscles induced by FOS ingestion (Tables 1, 4, and 6). The effect of FOS ingestion on the abundance of proteins composing the extracellular exosome was also observed in Sol muscles of mice raised under A1-*g* on the ISS (Supplementary Tables 7 and 8). These results suggested that ingestion of FOS might affect the alteration of components or types of extracellular vesicles produced by skeletal muscles^47^, but the effect may be different in mice exposed to μ-*g* or A1-*g*. However, we could not determine whether the effect of FOS ingestion is beneficial for skeletal muscles in this study.

### The proteome of Sol muscles in mice exposed to μ-*g* was also affected by gravitational reloading

Activation of blood coagulation and innate immune response was indicated in Sol muscles of mice exposed to μ-*g*, regardless of FOS ingestion (Table 8 and Supplementary Tables 3a and 4a), which had not been reported in previous studies^16,37^. In addition to these biological processes, fibrinolysis is also reportedly activated in response to skeletal muscle injury and has important role in the initial phase of repair^48–52^. However, negative regulation of fibrinolysis was indicated, especially in Sol muscles of mice exposed to μ-*g* without FOS ingestion, by the significant increase in Apoh, Plg, Serpinf2, and Hrg (Table 8 and Supplementary Table 3a). This result could be attributed to differences in the time of activation of these processes. One of the important roles of fibrinolysis is to remove fibrin deposited after blood coagulation for subsequent tissue repair, thus blood coagulation precedes the activation of fibrinolysis^48^. These results suggested that Sol muscles of mice exposed to μ-*g* were injured by gravitational reloading.

**Table 8:** Responses of soleus (Sol) muscle proteins involved in blood coagulation, fibrinolysis, innate immune response, and complement activation to microgravity (µ-*g*) or artificial 1-*g* (A1-*g*) exposure and/or fructo-oligosaccharide (FOS) ingestion.

Gene symbol (accession)	µ-*g* & FOS (−)/A1-*g* & FOS (−)	µ-*g* & FOS (+)/A1-*g* & FOS (−)	A1-*g* & FOS (+)/A1-*g* & FOS (−)
Blood coagulation & fibrinolysis			
Apoh (Q01339)	* FC = 4.97, *P* = 1.4E−4	* FC = 6.37, *P* = 1.0E−4	FC = 0.74, *P* = 8.3E−2
F12 (Q80YC5)	FC = 2.09, *P* = 1.1E−2	* FC = 3.67, *P* = 6.8E−4	FC = 0.79, *P* = 3.0E−1
Fga (E9PV24)	* FC = 3.46, *P* = 9.0E−3	* FC = 3.49, *P* = 8.9E−3	FC = 0.68, *P* = 3.6E−1
Fgb (Q8K0E8)	* FC = 3.36, *P* = 2.8E−3	* FC = 3.45, *P* = 2.2E−3	FC = 1.02, *P* = 8.4E−1
Fgg (Q8VCM7)	FC = 3.81, *P* = 1.1E−2	* FC = 4.08, *P* = 8.6E−3	FC = 0.80, *P* = 6.3E−1
Hspb1 (P14602)	* FC = 1.59, *P* = 1.3E−3	* FC = 1.80, *P* = 2.7E−3	FC = 1.13, *P* = 1.1E−1
Hrg (Q9ESB3)	* FC = 1.95, *P* = 7.6E−3	FC = 2.89, *P* = 1.1E−2	FC = 1.02, *P* = 8.2E−1
Kng1 (O08677)	* FC = 3.38, *P* = 3.3E−4	* FC = 4.72, *P* = 1.4E−3	FC = 0.83, *P* = 2.9E−1
Plg (P20918)	* FC = 6.06, *P* = 2.5E−4	* FC = 8.12, *P* = 3.5E−4	FC = 0.99, *P* = 9.6E−1
Serpinc1 (P32261)	FC = 2.49, *P* = 1.1E−2	* FC = 2.46, *P* = 5.1E−3	FC = 0.77, *P* = 2.9E−1
Serpind1 (P49182)	* FC = 5.84, *P* = 2.6E−4	* FC = 7.81, *P* = 3.2E−4	FC = 0.69, *P* = 1.2E−1
Serpinf2 (Q61247)	* FC = 3.07, *P* = 1.3E−3	* FC = 3.64, *P* = 3.8E−3	FC = 0.89, *P* = 5.0E−1
Serping1 (P97290)	* FC = 2.55, *P* = 6.0E−3	* FC = 3.72, *P* = 2.6E−3	FC = 0.74, *P* = 3.7E−1
Innate immune response & complement activation			
Ahsg (P29699)	* FC = 5.24, *P* = 1.5E−4	* FC = 7.18, *P* = 4.4E−4	FC = 0.80, *P* = 1.9E−1
Agt (P11859)	* FC = 8.79, *P* = 3.4E−4	* FC = 14.66, *P* = 1.3E−4	FC = 0.94, *P* = 8.3E−1
Anxa1 (P10107)	* FC = 2.78, *P* = 6.7E−4	* FC = 2.50, *P* = 6.4E−3	FC = 0.90, *P* = 4.2E−1
Anxa11 (P97384)	FC = 1.13, *P* = 3.9E−2	* FC = 1.26, *P* = 2.7E−3	FC = 1.04, *P* = 3.1E−1
C3 (P01027)	* FC = 4.48, *P* = 4.8E−4	* FC = 5.31, *P* = 7.5E−4	FC = 1.02, *P* = 9.0E−1
C4b (P01029)	* FC = 3.69, *P* = 3.1E−4	* FC = 5.06, *P* = 2.6E−4	FC = 0.72, *P* = 1.1E−1
C8g (Q8VCG4)	* FC = 3.60, *P* = 4.9E−4	* FC = 3.95, *P* = 3.8E−3	FC = 0.81, *P* = 4.1E−1
Cfh (P06909)	* FC = 3.04, *P* = 2.5E−3	* FC = 4.46, *P* = 1.3E−3	FC = 1.07, *P* = 9.7E−1
Cfi (Q61129)	* FC = 3.27, *P* = 1.2E−3	* FC = 4.54, *P* = 2.5E−3	FC = 0.82, *P* = 3.6E−1
Cfb (P04186)	* FC = 1.98, *P* = 2.8E−3	* FC = 2.22, *P* = 2.8E−3	FC = 0.97, *P* = 8.1E−1
Ighg1 (P01868)	FC = 5.87, *P* = 1.7E−2	* FC = 6.29, *P* = 6.1E−4	FC = 2.33, *P* = 1.7E−1

The protein abundance profiles in Sol muscles of the groups exposed to µ-*g* with and without FOS ingestion and the group exposed to A1-*g* with FOS ingestion were individually compared with that of the group exposed to A1-*g* without FOS ingestion. The results for the proteins, which were categorized as the related factors to blood coagulation fibrinolysis, innate immune response, and/or complement activation by DAVID Bioinformatics Resources 6.8 and significantly (*p* < 0.01) affected by µ-*g* exposure and/or FOS ingestion, are indicated. The fold change (FC) and *p* value (*P*) of each protein are depicted. *: the significant high- or low-abundance proteins.

It has been reported that gravitational reloading upon return to Earth causes histological damage to antigravity muscles of space-flown rats, including the Sol and adductor longus^53,54^. Similar phenomena were observed in Sol muscles of gravitational reloaded mice following hindlimb suspension^55,56^. In this study, mice in the μ-*g* and A1-*g* groups were similarly exposed to hypergravity and terrestrial gravity following spaceflight^17^. However, only Sol muscles of mice exposed to μ-*g* were damaged due to their high susceptibility to gravitational reloading. Soleus muscles, as well as the sarcomere intervals in the muscle fibers, are passively shortened during gravitational unloading due to plantar flexion of the ankle joint, and the development of tension is inhibited in this slackened condition^44,57,58^. However, the ability to develop tension is gradually restored as the number of sarcomeres in a single muscle fiber decreases and sarcomere intervals are extended in response to continual gravitational unloading. The subsequent forced dorsiflexion of the ankle joint and weight-bearing induced by gravitational reloading result in hyper-stretching and/or eccentric contraction of Sol muscles, which consequently results in muscle damage^54,59^.

The significant changes in the abundance of proteins categorized as Z-disk components in Sol muscles of mice exposed to μ-*g* suggested that sarcomere remodeling was probably induced in the muscles (Supplementary Tables 3, 4, and 9). It is reasonable to consider that Sol muscles were injured by hyper-stretching and/or eccentric contraction in response to gravitational reloading following μ-*g* exposure. We also observed higher serum levels of creatine kinase M-type, a marker of skeletal muscle injury, in mice exposed to μ-*g* than in mice exposed to A1-*g* (data not shown). These results clearly indicated that the effect of gravitational reloading on the proteome of Sol muscles in mice exposed to μ-*g* cannot be eliminated, even by comparing data obtained from mice raised under μ-*g* and A1-*g* on the ISS. The effect may have been more pronounced in this study because mice were exposed to terrestrial 1-*g* for a longer period prior to dissection than in previous studies^12–16^.

### Study limitations

This study was performed under certain limitations, which are described hereafter. A maximum of only 12 mice can be raised on the ISS using the MARS. On the second mission using the MARS, which was organized by JAXA, 12 mice were randomly divided into two groups and individually raised under µ-*g* or A1-*g*, and 3 mice in each group were fed a diet containing 5% FOS. The skeletal muscles sampled from this mission were analyzed in this study. It is not feasible to collect additional Sol and EDL muscle samples from space-flown mice. Therefore, the effect of type II error could not be eliminated owing to the small sample size (*n* = 3/group).

During spaceflight, the mice in the µ-*g* group floated in each MARS habitat cage, of which capacity was 560 cm^3^, but the mice in the A1-*g* group used only the floor, 101 cm^2^, as their living space. Therefore, in addition to gravitational unloading and reloading, the different behavior and physical activity of mice in µ-*g* and A1-*g* groups due to the different living space within the cage may have affected the proteome of Sol and EDL muscles. Additionally, we revealed the effects of μ-*g* exposure and/or FOS ingestion on the proteome of Sol and EDL muscles in developing mice from 9 to 12 weeks old. The alteration of the homeostatic proteome of these muscles in adult mice needs to be investigated in the future. Furthermore, we could not perform transcardial perfusion before isolating the skeletal muscles, as the dissection protocol was strictly controlled due to space experiment restrictions. Therefore, the abundance profiles of the skeletal muscle proteins were also affected by proteins in the blood. The abundance profiles of proteins in skeletal muscles and blood need to be individually determined in the future.

## Conclusions

The protein abundance profiles in Sol and EDL muscles were altered in response to both µ-*g* exposure and FOS ingestion. However, our results indicate that ingesting a diet containing 5% FOS is insufficient to suppress the atrophy and fiber-phenotype-shift in antigravity Sol muscles during spaceflight. The shift in fiber phenotype from slow- to fast-twitch type was prominent in Sol muscles in response to µ-*g* exposure. Ingestion of FOS tended to mitigate the decrease in proteins involved in oxidative metabolism. However, in association with the shift of fiber phenotype, the changes in abundance of proteins composing sarcomere and affecting muscle contractile properties were significant even in mice fed a diet containing FOS. In contrast, the abundance of proteins involved in the mitochondrial tricarboxylic acid cycle increased in EDL muscles following μ-*g* exposure, and this response tended to be enhanced by FOS ingestion. Additionally, the abundance of blood microparticle and extracellular exosome proteins in Sol and EDL muscles was significantly altered by μ-*g* exposure, and the responses of several proteins were also affected by FOS ingestion. Therefore, FOS ingestion may have affected extracellular vesicle components produced by skeletal muscles. Furthermore, blood coagulation and innate immune response were activated in Sol muscles following μ-*g* exposure, which suggested injury by acute gravitational reloading. The study findings indicated that the effect of gravitational reloading on Sol muscles in mice exposed to μ-*g* cannot be eliminated, even comparing with the data obtained from mice raised under μ-*g* and A1-*g* conditions on the ISS.

## Methods

### Experimental animals

All of the experimental procedures were conducted in accordance with the Guide for the Care and Use of Laboratory Animals of the Japanese Physiological Society and the National Institutes of Health. This study was approved by the Committees on Animal Care and Use of JAXA (accreditation no.: 016-018), Yokohama City University (accreditation no.: T-A-15-005), National Aeronautics and Space Administration (accreditation no.: FLT-17-106), and Explora Biolabs (accreditation no.: EB15-010).

One hundred male C57BL/6J mice, aged 5 weeks, were purchased from Jackson Laboratory (Bar Harbor, ME, USA) and were acclimatized to using a flight-type water nozzle. Finally, 12 mice, aged 9 weeks, were selected on the basis of body weight, food and water intake, and health conditions, and were launched to the ISS via SpaceX-12. The mice were randomly divided into μ-*g* (*n* = 6) and A1-*g* (*n* = 6) groups and individually raised in a habitat cage (capacity: 560 cm^3^, floor area: 101 cm^2^) of the MARS under each gravitational condition for 30 days. The mouse in each cage was given *ad libitum* access to food and water. Three FOS (+) mice, which were randomly selected, in each group were fed a modified AIN-93G diet containing 5% FOS (Oriental Yeast Co., Ltd., Tokyo, Japan) beginning 10 days prior to the launch. The remaining 3 FOS (−) mice were fed an energy-equivalent diet containing 5% cellulose instead of FOS (Meiji Food Materia Co. Ltd., Tokyo, Japan). The screening protocol, raising conditions, and nutritional composition of modified AIN-93G diet with and without FOS were previously reported in detail^17^. The GC group comprised 6 mice, which were individually raised in the ground model of the MARS habitat and transportation cage units. Similar to the μ-*g* and A1-*g* groups, 3 mice in the GC group were fed a diet containing 5% FOS, while the remaining 3 mice were fed an energy-equivalent diet containing 5% cellulose.

### Sample collection

All space-flown mice in the μ-*g* (*n* = 6) and A1-*g* (*n* = 6) groups were dissected within 36.5 h after splashdown of the Dragon vehicle, in which the MARS transportation cage unit was loaded, in the Pacific Ocean off the coast of California, USA^17^. Venous blood was collected from the mice under isoflurane anesthesia. The mice were subsequently euthanized by exsanguination under isoflurane anesthesia, and various tissues, including the Sol and EDL muscles, were isolated. The wet weight of the Sol and EDL muscles was measured, and the samples were snap-frozen with liquid nitrogen and stored at –80 °C for subsequent analyses. The mice in the GC group (*n* = 6) were dissected in the aforementioned manner.

### Proteomic analysis

The abundance profiles of muscle proteins were individually determined by proteomic analysis using LC-MS/MS and label-free quantitation. Proteins in the Sol and EDL muscles of the left limbs were extracted in SUP-1 and SUP-2 by a two-step solubilization method, and analyzed using the procedure described in our previous study^36^. For each supernatant, the MS/MS data of the Sol (*n* = 12) and EDL (*n* = 12) muscle proteins in the μ-*g* and A1-*g* groups were integrated but analyzed as two separate experiments using the Progenesis QI for proteomics software (version 2.0; Nonlinear Dynamics, Newcastle, UK). The normalized abundance of the peptides in SUP-1 and SUP-2 was separately quantified, but the same peptides in each of the supernatants were analyzed in the Sol and EDL muscles. The normalized abundance of the peptides in SUP-1 and SUP-2 was summed for each muscle, as described in a previous study^60^. Analyzed samples were classified according to the abundance of 1,253 proteins using the Perseus software (http://www.perseus-framework.org). Biological interpretation analysis for the Sol and EDL muscle proteins significantly affected by μ-*g* exposure and/or FOS ingestion was performed using DAVID Bioinformatics Resources 6.8 (https://david.ncifcrf.gov/) and Ingenuity Pathway Analysis (version 60467501; Qiagen, Hilden, Germany).

### Statistical analysis

All data in Figs. 1 and 2 are presented as the mean ± SD. Owing to the small sample size in each group (*n* = 3), the effects of spaceflight and FOS ingestion on the body weight, and wet weight of the muscles were evaluated by Kruskal−Wallis and Dunn’s multiple comparison tests, using GraphPad Prism version 7.02 software (GraphPad Software, La Jolla, CA, USA). Differences with *p* values < 0.05 were considered statistically significant.

The effects of μ-*g* exposure and FOS ingestion on the abundance of proteins in Sol and EDL muscles (*n* = 3/group) were individually evaluated by ANOVA, performed using the Progenesis QI for proteomics software. For multiple comparisons, differences with *p* values < 0.01 were considered statistically significant.

### Reporting summary

Further information on research design is available in the Nature Research Reporting Summary linked to this article.

## Supplementary information

Supplementary information

Reporting Summary

## Data Availability

The data that support the findings of this study are available from the corresponding authors upon reasonable request. The raw MS data and files obtained after analyses were deposited in the ProteomeXchange Consortium (http://proteomecentral.proteomexchange.org) via the jPOST partner repository (https://jpostdb.org) with the data set identifier PXD021550.
