# Supplementary material for: Effects of microgravity exposure and fructo-oligosaccharide ingestion on the proteome of soleus and extensor digitorum longus muscles in developing mice
Source: NPJ Microgravity. 2021 Sep 17;7:34. doi: 10.1038/s41526-021-00164-6 (PMC8448765; doi:10.1038/s41526-021-00164-6)

Supplementary Tables

Supplementary Table 1: Results of gene ontology analysis for the proteins extracted in supernatant-1 (SUP-1) and supernatant-2 (SUP-2) from soleus (Sol) and extensor digitorum longus (EDL) muscles of mice. As a result of LC-MS/MS analysis, 1,019 and 655 proteins were identified in SUP-1 and SUP-2, respectively, and 402 proteins were identified in both supernatants. Gene ontology analysis was performed using DAVID Bioinformatics Resources 6.8. The top 10 cellular components of the identified proteins in SUP-1 (**a**) and SUP-2 (**b**) are depicted.

a: The proteins in SUP-1

**Term**	**No. of proteins**
Cytoplasm	572
Extracellular exosome	528
Mitochondrion	361
Nucleus	360
Cytosol	263
Extracellular space	149
Nucleoplasm	136
Mitochondrial inner membrane	131
Extracellular region	112
Myelin sheath	105

b: The proteins in SUP-2

**Term**	**No. of proteins**
Membrane	354
Cytoplasm	331
Extracellular exosome	309
Mitochondrion	228
Nucleus	228
Mitochondrial inner membrane	130
Cytosol	95
Cytoskeleton	83
Myelin sheath	80
Focal adhesion	78

Supplementary Table 2: Lists of particularly responsive proteins in soleus (Sol) and extensor digitorum longus (EDL) muscles to microgravity (μ-*g*) exposure and/or fructo-oligosaccharide (FOS) ingestion. The protein abundance profiles of the groups exposed to μ-*g* with and without FOS ingestion and the group exposed to artificial 1-*g* (A1-*g*) with FOS ingestion were individually compared with that of the group exposed to A1-*g* without FOS ingestion. The responsive proteins, which are selected based on *p* values (< 0.01) and fold changes (> 2.0), in Sol (**a** and **b**) and EDL (**c** and **d**) are depicted. Additionally, the significant high- and low-abundance of proteins ($p < 0.01$) are indicated in orange and blue, respectively. Any protein in the group exposed to A1-*g* with FOS ingestion did not meet these criteria.

a: The high-abundance of proteins in Sol muscles of mice exposed to μ-*g* exposure

Accession	Gene name	μ-*g* & FOS (−) /A1-*g* & FOS (−)		μ-*g* & FOS (+) /A1-*g* & FOS (−)	
		Fold change	*P* value	Fold change	*P* value
**The responsive proteins to μ-*g* exposure with and without FOS ingestion**					
Q8BGT5	Alanine aminotransferase 2 (Gpt2)	Infinity	9.3×10^{-9}	Infinity	4.5×10^{-8}
Q9DBD0	Inhibitor of carbonic anhydrase (Ica)	16.78	2.2×10^{-4}	19.79	2.8×10^{-4}
Q9D1A4	Ankyrin repeat and SOCS box protein 5 (Asb5)	13.75	1.6×10^{-3}	20.76	7.3×10^{-4}
P14231	Sodium/potassium-transporting ATPase subunit beta-2 (Atp1b2)	13.52	6.6×10^{-3}	8.84	7.3×10^{-4}
Q64726	Zinc-alpha-2-glycoprotein (Azgp1)	9.08	2.2×10^{-3}	6.95	9.0×10^{-4}

P11859	Angiotensinogen (Agt)	8.79	3.4×10^{-4}	14.66	1.3×10^{-4}
P19182	Interferon-related developmental regulator 1 (Ifrd1)	8.21	3.3×10^{-4}	10.16	6.7×10^{-5}
Q8BUZ1	Actin-binding Rho-activating protein (Abra)	7.10	2.4×10^{-4}	7.71	1.9×10^{-4}
P20918	Plasminogen (Plg)	6.06	2.5×10^{-4}	8.12	3.5×10^{-4}
P49182	Heparin cofactor 2 (Serpind1)	5.84	2.6×10^{-4}	7.81	3.2×10^{-4}
P06330	Ig heavy chain V region AC38 205.12	5.79	4.0×10^{-3}	9.68	2.2×10^{-3}
Q00724	Retinol-binding protein 4 (Rbp4)	5.69	1.4×10^{-3}	9.36	2.2×10^{-4}
P29699	Alpha-2-HS-glycoprotein (Ahsg)	5.24	1.5×10^{-4}	7.18	4.4×10^{-4}
P22599	Alpha-1-antitrypsin 1-2 (Serpina1b)	5.07	2.7×10^{-4}	6.43	5.9×10^{-4}
Q01339	Beta-2-glycoprotein 1 (Apoh)	4.97	1.4×10^{-4}	6.37	1.0×10^{-4}
P06728	Apolipoprotein A-IV (Apoa4)	4.82	4.8×10^{-4}	5.97	2.9×10^{-4}
Q5SX39	Myosin-4 (Myh4)	4.78	7.1×10^{-4}	3.95	2.4×10^{-3}
P07724	Serum albumin (Alb)	4.77	5.1×10^{-4}	6.58	1.0×10^{-3}
P21614	Vitamin D-binding protein (Gc)	4.62	7.3×10^{-4}	6.50	1.6×10^{-3}
Q61696	Heat shock 70 kDa protein 1A (Hspa1a)	4.58	3.3×10^{-3}	6.06	2.4×10^{-4}
P01027	Complement C3 (C3)	4.48	4.8×10^{-4}	5.31	7.5×10^{-4}
Q61147	Ceruloplasmin (Cp)	4.31	1.1×10^{-3}	6.19	9.1×10^{-4}
P07309	Transthyretin (Ttr)	4.28	2.0×10^{-3}	6.16	1.6×10^{-3}
Q07456	Protein AMBP (Ambp)	4.27	1.0×10^{-3}	5.46	2.4×10^{-3}
P07759	Serine protease inhibitor A3K (Serpina3k)	4.22	4.0×10^{-4}	5.16	5.9×10^{-4}
P23953	Carboxylesterase 1C (Ces1c)	4.17	3.3×10^{-4}	6.44	5.7×10^{-4}
Q61702	Inter-alpha-trypsin inhibitor heavy chain H1 (Itih1)	4.12	1.5×10^{-3}	6.02	2.3×10^{-5}
Q8BG71	tRNA wybutosine-synthesizing protein 2 homolog (Trmt12)	4.08	4.3×10^{-4}	2.58	8.1×10^{-3}
Q91X72	Hemopexin (Hpx)	4.08	7.5×10^{-3}	6.89	5.8×10^{-3}
P52430	Serum paraoxonase/arylesterase 1 (Pon1)	3.86	1.6×10^{-3}	5.20	8.9×10^{-4}
P29788	Vitronectin (Vtn)	3.85	2.6×10^{-3}	10.03	4.5×10^{-4}

Q921I1	Serotransferrin (Tf)	3.85	2.8×10^{-3}	4.94	4.3×10^{-3}
Q00897	Alpha-1-antitrypsin 1-4 (Serpina1d)	3.79	4.1×10^{-4}	4.31	3.9×10^{-4}
Q9JHG6	Calcipressin-1 (Rcan1)	3.75	2.5×10^{-5}	5.92	6.7×10^{-5}
P01029	Complement C4-B (C4b)	3.69	3.1×10^{-4}	5.06	2.6×10^{-4}
Q8VCG4	Complement component C8 gamma chain (C8g)	3.60	4.9×10^{-4}	3.95	3.8×10^{-3}
Q61703	Inter-alpha-trypsin inhibitor heavy chain H2 (Itih2)	3.58	4.0×10^{-3}	4.97	2.4×10^{-3}
P70665	Sialate O-acetylesterase (Siae)	3.57	9.7×10^{-5}	3.46	9.4×10^{-3}
E9PV24	Fibrinogen alpha chain (Fga)	3.46	9.0×10^{-3}	3.49	8.9×10^{-3}
Q9DBB9	Carboxypeptidase N subunit 2 (Cpn2)	3.42	6.3×10^{-3}	3.93	6.6×10^{-3}
O08677	Kininogen-1 (Kng1)	3.38	3.3×10^{-4}	4.72	1.4×10^{-3}
Q8K0E8	Fibrinogen beta chain (Fgb)	3.36	2.8×10^{-3}	3.45	2.2×10^{-3}
Q61129	Complement factor I (Cfi)	3.27	1.2×10^{-3}	4.54	2.5×10^{-3}
Q61247	Alpha-2-antiplasmin (Serpinf2)	3.07	1.3×10^{-3}	3.64	3.8×10^{-3}
P06909	Complement factor H (Cfh)	3.04	2.5×10^{-3}	4.46	1.3×10^{-3}
P28665	Murinoglobulin-1 (Mug1)	2.97	1.2×10^{-3}	4.25	1.1×10^{-3}
P46412	Glutathione peroxidase 3 (Gpx3)	2.89	9.4×10^{-4}	3.55	1.2×10^{-4}
P13707	Glycerol-3-phosphate dehydrogenase [NAD(+)], cytoplasmic (Gpd1)	2.87	2.4×10^{-4}	2.69	4.4×10^{-4}
P10107	Annexin A1 (Anxa1)	2.78	6.7×10^{-4}	2.50	6.4×10^{-3}
P40936	Indolethylamine N-methyltransferase (Inmt)	2.56	4.5×10^{-4}	2.82	7.9×10^{-4}
P97290	Plasma protease C1 inhibitor (Serping1)	2.55	6.0×10^{-3}	3.72	2.6×10^{-3}
P97737	Growth/differentiation factor 10 (Gdf10)	2.51	4.2×10^{-3}	2.78	2.4×10^{-3}
Q00623	Apolipoprotein A-I (Apoa1)	2.41	2.6×10^{-3}	2.72	2.6×10^{-3}
Q80XB4	Nebulin-related-anchoring protein (Nrap)	2.30	1.3×10^{-3}	2.42	5.5×10^{-4}
Q06890	Clusterin (Clu)	2.23	1.3×10^{-3}	3.05	1.6×10^{-3}

P63328	Serine/threonine-protein phosphatase 2B catalytic subunit alpha isoform (Ppp3ca)	2.16	4.7×10^{-4}	2.28	3.6×10^{-3}
Q99JI1	Musculoskeletal embryonic nuclear protein 1 (Mustn1)	2.00	9.2×10^{-3}	2.48	5.8×10^{-4}
**The responsive proteins to μ-*g* exposure without FOS ingestion**					
P01786	Ig heavy chain V region MOPC 47A	Infinity	1.2×10^{-3}	Infinity	1.2×10^{-1}
Q8VD04	GRIP1-associated protein 1 (Gripap1)	61.34	9.7×10^{-3}	46.63	1.2×10^{-2}
P09041	Phosphoglycerate kinase 2 (Pgk2)	3.04	3.7×10^{-3}	2.97	1.1×10^{-2}
O88990	Alpha-actinin-3 (Actn3)	2.66	3.7×10^{-5}	1.82	1.4×10^{-3}
Q91WS7	DEP domain-containing protein 7 (Depdc7)	2.46	7.0×10^{-3}	1.71	1.1×10^{-1}
Q9JHR7	Insulin-degrading enzyme (Ide)	2.32	6.0×10^{-3}	2.23	1.3×10^{-2}
Q9D2G5	Synaptojanin-2 (Synj2)	2.16	8.3×10^{-3}	2.13	2.5×10^{-2}
O55047	Serine/threonine-protein kinase tousled-like 2 (Tlk2)	2.13	3.2×10^{-3}	1.57	3.3×10^{-2}
P32848	Parvalbumin alpha (Pvalb)	2.05	4.1×10^{-3}	2.60	3.1×10^{-2}
Q91WP6	Serine protease inhibitor A3N (Serpina3n)	2.05	2.0×10^{-3}	1.76	2.3×10^{-2}
P07901	Heat shock protein HSP 90-alpha (Hsp90aa1)	2.03	3.0×10^{-3}	2.56	1.4×10^{-2}
**The responsive proteins to μ-*g* exposure with FOS ingestion**					
Q9CR42	Ankyrin repeat domain-containing protein 1 (Ankrd1)	8.64	1.1×10^{-2}	11.38	7.9×10^{-3}
P17879	Heat shock 70 kDa protein 1B (Hspa1b)	5.33	1.8×10^{-2}	7.76	4.5×10^{-3}
P01868	Ig gamma-1 chain C region secreted form (Ighg1)	5.87	1.7×10^{-2}	6.29	6.1×10^{-4}
Q8VCM7	Fibrinogen gamma chain (Fgg)	3.81	1.1×10^{-2}	4.08	8.6×10^{-3}
Q80YC5	Coagulation factor XII (F12)	2.09	1.1×10^{-2}	3.67	6.8×10^{-4}
P01644	Ig kappa chain V-V region HP R16.7	1.92	1.9×10^{-4}	2.85	7.8×10^{-3}

P70695	Fructose-1,6-bisphosphatase isozyme 2 (Fbp2)	2.46	1.5×10^{-2}	2.71	2.4×10^{-3}
Q9WVD5	Mitochondrial ornithine transporter 1 (Slc25a15)	2.08	1.6×10^{-2}	2.51	6.8×10^{-3}
P19157	Glutathione S-transferase P 1 (Gstp1)	1.82	9.9×10^{-4}	2.50	1.4×10^{-3}
P32261	Antithrombin-III (Serpinc1)	2.49	1.1×10^{-2}	2.46	5.1×10^{-3}
Q9QUR7	Peptidyl-prolyl cis-trans isomerase NIMA-interacting 1 (Pin1)	1.32	5.3×10^{-2}	2.30	6.8×10^{-4}
P04186	Complement factor B (Cfb)	1.98	2.8×10^{-3}	2.22	2.8×10^{-3}
P17563	Methanethiol oxidase (Selenbp1)	1.57	4.1×10^{-5}	2.18	6.4×10^{-4}
P10649	Glutathione S-transferase Mu 1 (Gstm1)	1.91	1.5×10^{-4}	2.02	3.4×10^{-4}

b: The low-abundance of proteins in Sol muscles of mice exposed to μ-*g* exposure

**Accession**	**Gene name**	**μ-*g* & FOS (−) /A1-*g* & FOS (−)**		**μ-*g* & FOS (+) /A1-*g* & FOS (−)**	
		**Fold change**	***P* value**	**Fold change**	***P* value**
**The responsive proteins to μ-*g* exposure with and without FOS ingestion**					
Q9R0H0	Peroxisomal acyl-coenzyme A oxidase 1 (Acox1)	0.15	8.0×10^{-4}	0.20	1.9×10^{-3}
Q80TJ7	Histone lysine demethylase PHF8 (Phf8)	0.18	1.1×10^{-3}	0.23	1.4×10^{-4}
P13705	DNA mismatch repair protein (Msh3)	0.21	2.2×10^{-4}	0.28	9.7×10^{-4}
Q9CRB8	Mitochondrial fission process protein 1 (Mtfp1)	0.22	1.0×10^{-3}	0.36	1.6×10^{-3}
Q80XN0	D-beta-hydroxybutyrate dehydrogenase, mitochondrial (Bdh1)	0.30	1.6×10^{-3}	0.40	6.1×10^{-3}
P13542	Myosin-8 (Myh8)	0.30	5.4×10^{-4}	0.36	4.4×10^{-4}
Q2TPA8	Hydroxysteroid dehydrogenase-like protein 2 (Hsdl2)	0.34	5.8×10^{-4}	0.44	1.5×10^{-3}
O88746	Target of Myb protein 1 (Tom1)	0.35	4.2×10^{-4}	0.43	5.3×10^{-4}

A2AQP0	Myosin-7B (Myh7b)	0.36	2.3×10^{-3}	0.44	2.5×10^{-3}
Q8CC35	Synaptopodin (Synpo)	0.37	1.6×10^{-4}	0.40	5.8×10^{-4}
P56213	FAD-linked sulfhydryl oxidase ALR (Gfer)	0.38	2.4×10^{-3}	0.33	1.8×10^{-3}
O35459	Delta(3,5)-Delta(2,4)-dienoyl-CoA isomerase, mitochondrial (Ech1)	0.38	6.1×10^{-4}	0.46	1.1×10^{-3}
Q5EBG6	Heat shock protein beta-6 (Hspb6)	0.38	5.7×10^{-4}	0.41	1.7×10^{-4}
Q8CI43	Myosin light chain 6B (Myl6b)	0.43	6.3×10^{-3}	0.47	3.6×10^{-3}
**The responsive proteins to μ-*g* exposure without FOS ingestion**					
P97355	Spermine synthase (Sms)	0.13	4.7×10^{-3}	2.09	9.4×10^{-1}
Q6W8Q3	Purkinje cell protein 4-like protein 1 (Pcp4l1)	0.15	4.2×10^{-4}	0.12	2.4×10^{-2}
Q9CQE8	RNA transcription, translation and transport factor protein (Rtraf)	0.30	8.8×10^{-3}	0.52	1.4×10^{-1}
Q99NB1	Acetyl-coenzyme A synthetase 2-like, mitochondrial (Acss1)	0.36	9.7×10^{-4}	0.54	5.4×10^{-3}
Q9R0Q7	Prostaglandin E synthase 3 (Ptges3)	0.40	5.1×10^{-3}	0.25	3.1×10^{-2}
Q8R3Q6	Coiled-coil domain-containing protein 58 (Ccdc58)	0.43	2.9×10^{-3}	0.53	1.9×10^{-2}
Q9CZR8	Elongation factor Ts, mitochondrial (Tsfm)	0.44	1.3×10^{-3}	0.70	4.2×10^{-2}
Q8K4G5	Actin-binding LIM protein 1 (Ablim1)	0.44	1.0×10^{-3}	0.52	1.1×10^{-2}
O35678	Monoglyceride lipase (Mgll)	0.44	2.0×10^{-3}	0.73	1.2×10^{-2}
Q8VCW8	Medium-chain acyl-CoA ligase ACSF2, mitochondrial (Acsf2)	0.45	7.7×10^{-4}	0.63	3.4×10^{-2}
Q9CYH2	Peroxiredoxin-like 2A (Prxl2a)	0.46	3.0×10^{-4}	0.73	4.1×10^{-2}
P34914	Bifunctional epoxide hydrolase 2 (Ephx2)	0.48	5.4×10^{-4}	0.60	9.9×10^{-4}
P23927	Alpha-crystallin B chain (Cryab)	0.48	1.1×10^{-3}	0.54	1.7×10^{-5}
Q99P30	Peroxisomal coenzyme A diphosphatase (Nudt7)	0.49	8.2×10^{-4}	0.53	1.0×10^{-2}
P24270	Catalase (Cat)	0.49	3.5×10^{-3}	0.62	1.0×10^{-2}
Q8VDN2	Sodium/potassium-transporting ATPase subunit alpha-1 (Atp1a1)	0.49	6.7×10^{-3}	0.55	1.6×10^{-2}

**The responsive proteins to μ-*g* exposure with FOS ingestion**					
Q61398	Procollagen C-endopeptidase enhancer 1 (Pcolce)	0.24	6.1×10^{-2}	0.12	4.9×10^{-3}
P43025	Tetranectin (Clec3b)	0.04	4.0×10^{-2}	0.23	3.7×10^{-3}
P08030	Adenine phosphoribosyltransferase (Aprt)	0.51	3.2×10^{-2}	0.37	4.1×10^{-3}
P62270	40S ribosomal protein S18 (Rps18)	0.29	3.7×10^{-2}	0.40	5.6×10^{-3}
Q9EQK5	Major vault protein (Mvp)	0.32	1.6×10^{-2}	0.40	6.6×10^{-3}
P63024	Vesicle-associated membrane protein 3 (Vamp3)	0.86	4.8×10^{-1}	0.42	4.4×10^{-3}
P27546	Microtubule-associated protein 4 (Map4)	0.53	3.5×10^{-2}	0.46	2.3×10^{-3}
Q62009	Periostin (Postn)	0.53	3.4×10^{-3}	0.46	4.9×10^{-3}
Q99LP6	GrpE protein homolog 1, mitochondrial (Grpel1)	0.51	4.3×10^{-2}	0.48	2.0×10^{-3}
Q9D5V5	Cullin-5 (Cul5)	0.59	9.9×10^{-4}	0.49	5.4×10^{-3}

c: The high-abundance of proteins in EDL muscles of mice exposed to μ-*g* exposure

**Accession**	**Gene name**	**μ-*g* & FOS (−) /A1-*g* & FOS (−)**		**μ-*g* & FOS (+) /A1-*g* & FOS (−)**	
		**Fold change**	***P* value**	**Fold change**	***P* value**
**The responsive proteins to μ-*g* exposure without FOS ingestion**					
O70571	[Pyruvate dehydrogenase (acetyl-transferring)] kinase isozyme 4, mitochondrial (Pdk4)	2.82	8.7×10^{-3}	3.42	1.0×10^{-2}
**The responsive proteins to μ-*g* exposure with FOS ingestion**					
Q7TSI1	Pleckstrin homology domain-containing family M member 1 (Plekhm1)	Infinity	1.2×10^{-1}	Infinity	4.8×10^{-7}
Q9DBB9	Carboxypeptidase N subunit 2 (Cpn2)	1.00	1.0	Infinity	3.4×10^{-5}
Q9R0H0	Peroxisomal acyl-coenzyme A oxidase 1 (Acox1)	2.26	2.7×10^{-2}	3.70	8.1×10^{-3}
O89086	RNA-binding protein 3 (Rbm3)	1.97	5.5×10^{-4}	2.30	1.7×10^{-3}

Q69ZT9	TBC1 domain family member 30 (Tbc1d30)	1.39	1.1×10^{-1}	2.23	1.2×10^{-3}
P13705	DNA mismatch repair protein Msh3 (Msh3)	1.90	4.4×10^{-3}	2.16	2.8×10^{-3}

d: The low-abundance of proteins in EDL muscles of mice exposed to μ-*g* exposure

**Accession**	**Gene name**	**μ-*g* & FOS (−) /A1-*g* & FOS (−)**		**μ-*g* & FOS (+) /A1-*g* & FOS (−)**	
		**Fold change**	***P* value**	**Fold change**	***P* value**
**The responsive proteins to μ-*g* exposure with and without FOS ingestion**					
P11680	Properdin (Cfp)	0.002	1.4×10^{-3}	0.001	1.4×10^{-3}
**The responsive proteins to μ-*g* exposure without FOS ingestion**					
Q8BTM8	Filamin-A (Flna)	Infinity	4.8×10^{-5}	0.71	4.1×10^{-1}
P47911	60S ribosomal protein L6 (Rpl6)	0.46	8.6×10^{-4}	0.53	1.5×10^{-2}
**The responsive proteins to μ-*g* exposure with FOS ingestion**					
Q8VED5	Keratin, type II cytoskeletal 79 (Krt79)	1.04	5.5×10^{-1}	0.06	2.5×10^{-3}
Q8R010	Aminoacyl tRNA synthase complex-interacting multifunctional protein 2 (Aimp2)	0.58	5.1×10^{-4}	0.44	1.4×10^{-3}
P80318	T-complex protein 1 subunit gamma (Cct3)	0.61	1.3×10^{-2}	0.45	2.8×10^{-3}
Q8BML9	Glutamine--tRNA ligase (Qars)	0.58	2.5×10^{-3}	0.48	4.1×10^{-4}
P31230	Aminoacyl tRNA synthase complex-interacting multifunctional protein 1 (Aimp1)	0.65	1.4×10^{-2}	0.49	2.9×10^{-3}

Supplementary Table 3: Results of gene ontology analysis for the soleus (Sol) muscle proteins, which were significantly affected by microgravity (μ-*g*) exposure.

Gene ontology analysis was performed using DAVID Bioinformatics Resources 6.8. The results for the significant higher- (**a**) and lower-abundance (**b**) of proteins ($p < 0.01$) in Sol muscles of mice exposed to μ-*g* without fructo-oligosaccharide (FOS) ingestion than those of mice exposed to artificial 1-*g* (A1-*g*) without FOS ingestion are depicted. The indices in biological process and cellular component with Benjamini values less than 0.05 are shown.

a: For the high-abundance of proteins in μ-*g* & FOS (−) vs. A1-*g* & FOS (−) groups

**Term**	**No. of proteins**	**Benjamini value**
**Biological process**		
Acute-phase response	5	8.80×10^{-3}
Blood coagulation	9	5.50×10^{-5}
Carbohydrate metabolic process	11	3.60×10^{-4}
Complement activation, alternative pathway	4	3.50×10^{-3}
Complement activation, classical pathway	5	3.50×10^{-2}
Fibrinolysis	5	4.20×10^{-4}
Gluconeogenesis	6	1.80×10^{-4}
Glutathione metabolic process	6	2.70×10^{-3}
Glycolytic process	7	5.20×10^{-5}
Hemostasis	7	1.90×10^{-4}
Metabolic process	12	3.00×10^{-2}
Muscle contraction	5	2.90×10^{-2}
Negative regulation of fibrinolysis	4	1.40×10^{-3}
Negative regulation of peptidase activity	12	8.50×10^{-7}

Organ regeneration	5	3.50×10^{-2}
Oxidation-reduction process	21	4.30×10^{-5}
Phosphorylation	14	2.90×10^{-2}
Positive regulation of fast-twitch skeletal muscle fiber contraction	3	1.10×10^{-2}
Response to peptide hormone	6	1.20×10^{-2}
Skeletal muscle fiber development	5	3.60×10^{-3}
**Cellular component**		
Blood microparticle	33	1.00×10^{-38}
Cell surface	13	1.50×10^{-2}
Chylomicron	3	3.40×10^{-2}
Cytoplasm	78	1.30×10^{-6}
Cytosol	30	3.00×10^{-4}
Extracellular exosome	86	4.10×10^{-38}
Extracellular matrix	11	9.50×10^{-4}
Extracellular region	48	5.40×10^{-15}
Extracellular space	56	1.00×10^{-24}
Extracellular vesicle	5	5.50×10^{-3}
Extrinsic component of external side of plasma membrane	3	1.50×10^{-2}
Fibrinogen complex	3	1.20×10^{-2}
Glycerol-3-phosphate dehydrogenase complex	3	2.30×10^{-3}
High-density lipoprotein particle	4	5.50×10^{-3}
Mitochondrion	28	9.40×10^{-4}
Myelin sheath	9	1.10×10^{-3}
Myosin filament	3	3.40×10^{-2}
Protein complex	17	2.40×10^{-4}
Sarcomere	5	2.20×10^{-3}
Sarcoplasmic reticulum	5	9.10×10^{-3}
Sperm fibrous sheath	3	3.80×10^{-2}
Spherical high-density lipoprotein particle	3	1.50×10^{-2}
Z disc	6	1.80×10^{-2}

b: For the low-abundance of proteins in μ-*g* & FOS (−) vs. A1-*g* & FOS (−) groups

**Term**	**No. of proteins**	**Benjamini value**

**Biological process**		
2-oxoglutarate metabolic process	5	1.90×10^{-3}
Fatty acid beta-oxidation	15	1.90×10^{-15}
Fatty acid beta-oxidation using acyl-CoA dehydrogenase	6	1.00×10^{-4}
Fatty acid metabolic process	20	3.40×10^{-13}
Lipid homeostasis	5	4.20×10^{-2}
Lipid metabolic process	21	4.40×10^{-6}
Metabolic process	26	1.20×10^{-9}
Mitochondrial electron transport, cytochrome c to oxygen	5	3.50×10^{-4}
NADH metabolic process	4	1.30×10^{-2}
Oxidation-reduction process	41	2.70×10^{-17}
Respiratory electron transport chain	4	4.50×10^{-2}
Transport	41	1.20×10^{-4}
Tricarboxylic acid cycle	10	9.80×10^{-10}
**Cellular component**		
Actin cytoskeleton	10	2.50×10^{-3}
Contractile fiber	4	1.10×10^{-2}
Extracellular exosome	86	5.60×10^{-24}
Extracellular matrix	11	9.20×10^{-3}
Focal adhesion	18	5.70×10^{-6}
Mitochondrial inner membrane	46	1.10×10^{-33}
Mitochondrial intermembrane space	9	1.20×10^{-5}
Mitochondrial matrix	27	5.60×10^{-21}
Mitochondrial nucleoid	6	1.40×10^{-3}
Mitochondrial respiratory chain complex I	11	7.00×10^{-10}
Mitochondrial respiratory chain complex IV	6	5.10×10^{-6}
Mitochondrion	106	2.10×10^{-58}
Myelin sheath	23	6.30×10^{-16}
Peroxisome	14	1.30×10^{-8}
Pyruvate dehydrogenase complex	3	3.40×10^{-2}
Respiratory chain	14	3.50×10^{-13}
Z disc	12	8.70×10^{-7}

Supplementary Table 4: Results of gene ontology analysis for the soleus (Sol) muscle proteins, which were significantly affected by microgravity (μ-*g*) exposure and fructo-oligosaccharide (FOS) ingestion. Gene ontology analysis was performed using DAVID Bioinformatics Resources 6.8. The results for the significant higher- (**a**) and lower-abundance (**b**) of proteins ($p < 0.01$) in Sol muscles of mice exposed to μ-*g* with FOS ingestion than those of mice exposed to artificial 1-*g* (A1-*g*) without FOS ingestion are depicted. The indices in biological process and cellular component with Benjamini values less than 0.05 are shown.

a: For the high-abundance of proteins in μ-*g* & FOS (+) vs. A1-*g* & FOS (−) groups

**Term**	**No. of proteins**	**Benjamini value**
**Biological process**		
Blood coagulation	11	1.20×10^{-6}
Blood coagulation, fibrin clot formation	3	3.70×10^{-2}
Cellular detoxification of nitrogen compound	3	1.50×10^{-2}
Cellular response to drug	6	2.50×10^{-2}
Complement activation, alternative pathway	4	5.80×10^{-3}
Complement activation, classical pathway	6	7.40×10^{-3}
Fibrinolysis	6	2.20×10^{-5}
Gluconeogenesis	6	3.70×10^{-4}
Glutathione metabolic process	9	1.60×10^{-6}
Hemostasis	9	1.20×10^{-6}
Innate immune response	12	2.70×10^{-2}
Negative regulation of peptidase activity	12	1.20×10^{-6}
Nitrobenzene metabolic process	3	2.50×10^{-2}
Oxidation-reduction process	19	1.30×10^{-3}

Plasminogen activation	4	5.80×10^{-3}
Positive regulation of fast-twitch skeletal muscle fiber contraction	3	1.50×10^{-2}
Protein polymerization	5	4.10×10^{-4}
Response to calcium ion	6	1.70×10^{-2}
Skeletal muscle fiber development	6	3.80×10^{-4}
Xenobiotic catabolic process	4	2.10×10^{-3}
**Cellular component**		
Blood microparticle	33	1.50×10^{-37}
Cell cortex	6	4.40×10^{-2}
Cell surface	14	1.20×10^{-2}
Chylomicron	3	4.20×10^{-2}
Cytoplasm	86	1.90×10^{-7}
Cytosol	37	1.40×10^{-6}
Extracellular exosome	91	2.80×10^{-38}
Extracellular matrix	12	3.50×10^{-4}
Extracellular region	54	9.20×10^{-18}
Extracellular space	61	7.90×10^{-27}
Extracellular vesicle	6	6.00×10^{-4}
Extrinsic component of external side of plasma membrane	3	1.70×10^{-2}
Fibrinogen complex	4	3.50×10^{-4}
Glycerol-3-phosphate dehydrogenase complex	3	2.40×10^{-3}
High-density lipoprotein particle	4	6.50×10^{-3}
I band	5	6.00×10^{-4}
Mitochondrion	30	6.00×10^{-4}
Myelin sheath	9	1.70×10^{-3}
Myofibril	5	3.20×10^{-3}
Neuron projection	10	4.40×10^{-2}
Platelet alpha granule	3	4.90×10^{-2}
Protein complex	18	1.60×10^{-4}
Sarcomere	4	2.90×10^{-2}
Sarcoplasmic reticulum	6	1.30×10^{-3}
Spherical high-density lipoprotein particle	3	1.70×10^{-2}
Z disc	10	1.40×10^{-5}

b: For the low-abundance of proteins in μ-*g* & FOS (+) vs. A1-*g* & FOS (−) groups

**Term**	**No. of proteins**	**Benjamini value**
**Biological process**		
Carnitine metabolic process, CoA-linked	3	5.10×10^{-3}
Fatty acid beta-oxidation	9	3.50×10^{-8}
Fatty acid beta-oxidation using acyl-CoA dehydrogenase	5	2.70×10^{-4}
Fatty acid metabolic process	11	2.50×10^{-6}
Lipid homeostasis	5	4.50×10^{-3}
Lipid metabolic process	12	2.60×10^{-3}
Metabolic process	16	3.40×10^{-6}
Muscle filament sliding	3	2.80×10^{-2}
Oxidation-reduction process	16	2.70×10^{-4}
Regulation of cholesterol metabolic process	3	4.40×10^{-2}
Tricarboxylic acid cycle	4	2.60×10^{-2}
**Cellular component**		
Axon	8	3.90×10^{-2}
Contractile fiber	3	4.90×10^{-2}
Cytoplasm	59	3.50×10^{-5}
Extracellular exosome	44	9.50×10^{-11}
Extracellular matrix	8	1.40×10^{-2}
Focal adhesion	10	3.50×10^{-3}
Mitochondrial inner membrane	17	8.00×10^{-9}
Mitochondrial matrix	13	1.00×10^{-8}
Mitochondrion	42	1.00×10^{-15}
Myelin sheath	9	1.60×10^{-4}
Myosin complex	4	3.30×10^{-2}
Myosin filament	3	2.90×10^{-2}
Peroxisome	7	1.20×10^{-3}
Z disc	9	8.50×10^{-6}

Supplementary Table 5: Results of gene ontology analysis for the extensor digitorum longus (EDL) muscle proteins, which were significantly affected by microgravity (μ-*g*) exposure. Gene ontology analysis was performed using DAVID Bioinformatics Resources 6.8. The results for the significant higher- (**a**) and lower-abundance (**b**) of proteins ($p < 0.01$) in EDL muscles of mice exposed to μ-*g* without fructo-oligosaccharide (FOS) ingestion than those of mice exposed to artificial 1-*g* (A1-*g*) without FOS ingestion are depicted. The indices in biological process and cellular component with Benjamini values less than 0.05 are shown.

a: For the high-abundance of proteins in μ-*g* & FOS (−) vs. A1-*g* & FOS (−) groups

**Term**	**No. of proteins**	**Benjamini value**
**Biological process**		
Metabolic process	6	3.70×10^{-2}
Oxidation-reduction process	11	2.00×10^{-6}
Tricarboxylic acid cycle	3	4.80×10^{-2}
**Cellular component**		
Extracellular exosome	12	6.20×10^{-3}
Mitochondrial matrix	4	2.80×10^{-2}
Mitochondrion	11	2.20×10^{-3}
Myelin sheath	5	3.30×10^{-3}

b: For the low-abundance of proteins in μ-*g* & FOS (−) vs. A1-*g* & FOS (−) groups

**Term**	**No. of proteins**	**Benjamini value**
**Biological process**		
Cytoplasmic translation	4	7.00×10^{-3}

Translation	10	2.70×10^{-5}
Translational elongation	4	1.20×10^{-2}
**Cellular component**		
Cell-cell adherens junction	6	4.10×10^{-3}
Cytoplasm	27	1.30×10^{-4}
Cytoplasmic ribonucleoprotein granule	3	1.30×10^{-2}
Cytosol	14	2.10×10^{-4}
Cytosolic large ribosomal subunit	5	3.90×10^{-4}
Eukaryotic translation elongation factor 1 complex	2	4.30×10^{-2}
Extracellular exosome	21	4.20×10^{-7}
Extracellular matrix	9	2.50×10^{-6}
Focal adhesion	11	2.30×10^{-7}
Intracellular	10	2.20×10^{-2}
Intracellular ribonucleoprotein complex	8	4.80×10^{-5}
Melanosome	4	1.00×10^{-2}
Myelin sheath	4	4.60×10^{-2}
Nucleolus	7	4.00×10^{-2}
Nucleus	22	1.10×10^{-2}
Protein complex	6	4.40×10^{-2}
Ribosome	7	4.00×10^{-5}

Supplementary Table 6: Results of gene ontology analysis for the extensor digitorum longus (EDL) muscle proteins, which were significantly affected by microgravity (μ-*g*) exposure and fructo-oligosaccharide (FOS) ingestion. Gene ontology analysis was performed using DAVID Bioinformatics Resources 6.8. The results for the significant higher- (**a**) and lower-abundance (**b**) of proteins ($p < 0.01$) in EDL muscles of mice exposed to μ-*g* with FOS ingestion than those of mice exposed to artificial 1-*g* (A1-*g*) without FOS ingestion are depicted. The indices in biological process and cellular component with Benjamini values less than 0.05 are shown.

a: For the high-abundance of proteins in μ-*g* & FOS (+) vs. A1-*g* & FOS (−) groups

**Term**	**No. of proteins**	**Benjamini value**
**Biological process**		
Oxidation-reduction process	12	7.20×10^{-4}
Tricarboxylic acid cycle	4	1.20×10^{-2}
**Cellular component**		
Blood microparticle	5	1.40×10^{-2}
Cytoplasm	34	4.40×10^{-3}
Extracellular exosome	27	5.10×10^{-7}
Mitochondrion	19	5.10×10^{-5}
Myelin sheath	8	5.10×10^{-5}

b: For the low-abundance of proteins in μ-*g* & FOS (+) vs. A1-*g* & FOS (−) groups

**Term**	**No. of proteins**	**Benjamini value**
**Biological process**		
Protein folding	5	4.20×10^{-2}

Translation	15	1.50×10^{-9}
Translational elongation	5	1.10×10^{-3}
tRNA aminoacylation for protein translation	5	5.60×10^{-4}
**Cellular component**		
Aminoacyl-tRNA synthetase multienzyme complex	4	2.80×10^{-5}
Cell body	4	2.40×10^{-2}
Cell-cell adherens junction	8	4.60×10^{-4}
Cytoplasm	36	4.90×10^{-5}
Cytosol	22	1.40×10^{-7}
Cytosolic small ribosomal subunit	4	5.60×10^{-3}
Extracellular exosome	29	1.70×10^{-9}
Extracellular matrix	8	3.40×10^{-4}
Focal adhesion	8	1.50×10^{-3}
Intracellular ribonucleoprotein complex	7	3.40×10^{-3}
Melanosome	4	2.40×10^{-2}
Mitochondrion	13	1.90×10^{-2}
Myelin sheath	5	1.90×10^{-2}
Polysome	3	4.80×10^{-2}
Ribosome	5	1.90×10^{-2}
Small ribosomal subunit	3	2.40×10^{-2}

Supplementary Table 7: Responses of soleus (Sol) and extensor digitorum longus (EDL) muscle proteins categorized as the components of blood microparticle, extracellular exosome, and extracellular vesicle to microgravity (μ-*g*) or artificial 1-*g* (A1-*g*) exposure and/or fructo-oligosaccharide (FOS) ingestion. The protein abundance profiles of the groups exposed to μ-*g* with and without FOS ingestion and the group exposed to A1-*g* with FOS ingestion were individually compared with that of the group exposed to A1-*g* without FOS ingestion. The results for the proteins, which were categorized as the components of blood microparticle, extracellular exosome, and/or extracellular vesicle by DAVID Bioinformatics Resources 6.8 and significantly ($p < 0.01$) affected by μ-*g* exposure and/or FOS ingestion, in Sol (**a**) and EDL (**b**) muscles are indicated. The significant high- and low-abundance of proteins are indicated in orange and blue, respectively.

a: The proteins in Sol muscles

**Accession**	**Gene name**	**μ-*g* & FOS (−) /A1-*g* & FOS (−)**		**μ-*g* & FOS (+) /A1-*g* & FOS (−)**		**A1-*g* & FOS (+) /A1-*g* & FOS (−)**	
		**Fold change**	***P* value**	**Fold change**	***P* value**	**Fold change**	***P* value**
Q8VD04	GRIP1 associated protein 1 (Gripap1)	61.34	9.7×10^{-3}	46.63	1.2×10^{-2}	2.12	8.5×10^{-1}
Q64726	Alpha-2-glycoprotein 1, zinc (Azgp1)	9.08	2.2×10^{-3}	6.95	9.0×10^{-4}	1.41	6.9×10^{-1}

P11859	Angiotensinogen (serpin peptidase inhibitor, clade A, member 8) (Agt)	8.79	3.4×10^{-4}	14.66	1.3×10^{-4}	0.94	8.3×10^{-1}
P20918	Plasminogen (Plg)	6.06	2.5×10^{-4}	8.12	3.5×10^{-4}	0.99	9.6×10^{-1}
P49182	Serine (or cysteine) peptidase inhibitor, clade D, member 1 (Serpind1)	5.84	2.6×10^{-4}	7.81	3.2×10^{-4}	0.69	1.2×10^{-1}
Q00724	Retinol binding protein 4, plasma (Rbp4)	5.69	1.4×10^{-3}	9.36	2.2×10^{-4}	0.92	7.3×10^{-1}
P29699	Alpha-2-HS-glycoprotein (Ahsg)	5.24	1.5×10^{-4}	7.18	4.4×10^{-4}	0.80	1.9×10^{-1}
P22599	Serine (or cysteine) preptidase inhibitor, clade A, member 1B (Serpina1b)	5.07	2.7×10^{-4}	6.43	5.9×10^{-4}	0.88	4.5×10^{-1}
Q01339	Apolipoprotein H (Apoh)	4.97	1.4×10^{-4}	6.37	1.0×10^{-4}	0.74	8.3×10^{-2}
P06728	Apolipoprotein A-IV (Apoa4)	4.82	4.8×10^{-4}	5.97	2.9×10^{-4}	0.64	5.9×10^{-2}
P07724	Albumin (Alb)	4.77	5.1×10^{-4}	6.58	1.0×10^{-3}	0.99	9.9×10^{-1}
P21614	Group specific component (Gc)	4.62	7.3×10^{-4}	6.50	1.6×10^{-3}	0.85	3.8×10^{-1}
Q61696	Heat shock protein 1A (Hspa1a)	4.58	3.3×10^{-3}	6.06	2.4×10^{-4}	1.12	4.6×10^{-1}
P01027	Complement component 3 (C3)	4.48	4.8×10^{-4}	5.31	7.5×10^{-4}	1.02	9.0×10^{-1}
Q61147	Ceruloplasmin (Cp)	4.31	1.1×10^{-3}	6.19	9.1×10^{-4}	1.01	8.8×10^{-1}
P07309	Transthyretin (Ttr)	4.28	2.0×10^{-3}	6.16	1.6×10^{-3}	0.87	6.7×10^{-1}
Q07456	Alpha 1 microglobulin/bikunin (Ambp)	4.27	1.0×10^{-3}	5.46	2.4×10^{-3}	0.93	7.8×10^{-1}
P07759	Serine (or cysteine) peptidase inhibitor, clade A, member 3K (Serpina3k)	4.22	4.0×10^{-4}	5.16	5.9×10^{-4}	0.79	3.2×10^{-1}
Q61702	Inter-alpha trypsin inhibitor, heavy chain 1 (Itih1)	4.12	1.5×10^{-3}	6.02	2.3×10^{-5}	0.86	4.8×10^{-1}
Q91X72	Hemopexin (Hpx)	4.08	7.5×10^{-3}	6.89	5.8×10^{-3}	0.97	9.3×10^{-1}
P52430	Paraoxonase 1 (Pon1)	3.86	1.6×10^{-3}	5.20	8.9×10^{-4}	0.89	6.5×10^{-1}
P29788	Vitronectin (Vtn)	3.85	2.6×10^{-3}	10.03	4.5×10^{-4}	1.10	6.9×10^{-1}
Q921I1	Transferrin (Trf)	3.85	2.8×10^{-3}	4.94	4.3×10^{-3}	0.93	8.3×10^{-1}
Q00897	Serine (or cysteine) peptidase inhibitor, clade A, member 1D (Serpina1d)	3.79	4.1×10^{-4}	4.31	3.9×10^{-4}	0.92	6.0×10^{-1}

P01029	Complement component 4B (Chido blood group) (C4b)	3.69	3.1×10^{-4}	5.06	2.6×10^{-4}	0.72	1.1×10^{-1}
Q8VCG4	Complement component 8, gamma polypeptide (C8g)	3.60	4.9×10^{-4}	3.95	3.8×10^{-3}	0.81	4.1×10^{-1}
Q61703	Inter-alpha trypsin inhibitor, heavy chain 2 (Itih2)	3.58	4.0×10^{-3}	4.97	2.4×10^{-3}	0.98	9.6×10^{-1}
P70665	Sialic acid acetylesterase (Siae)	3.57	9.7×10^{-5}	3.46	9.4×10^{-3}	0.79	2.6×10^{-1}
E9PV24	Fibrinogen alpha chain (Fga)	3.46	9.0×10^{-3}	3.49	8.9×10^{-3}	0.68	3.6×10^{-1}
Q9DBB9	Carboxypeptidase N, polypeptide 2 (Cpn2)	3.42	6.3×10^{-3}	3.93	6.6×10^{-3}	0.66	3.3×10^{-1}
O08677	Kininogen 1 (Kng1)	3.38	3.3×10^{-4}	4.72	1.4×10^{-3}	0.83	2.9×10^{-1}
Q8K0E8	Fibrinogen beta chain (Fgb)	3.36	2.8×10^{-3}	3.45	2.2×10^{-3}	1.02	8.4×10^{-1}
Q61129	Complement component factor I (Cfi)	3.27	1.2×10^{-3}	4.54	2.5×10^{-3}	0.82	3.6×10^{-1}
Q61247	Serine (or cysteine) peptidase inhibitor, clade F, member 2 (Serpinf2)	3.07	1.3×10^{-3}	3.64	3.8×10^{-3}	0.89	5.0×10^{-1}
P09041	Phosphoglycerate kinase 2 (Pgk2)	3.04	3.7×10^{-3}	2.97	1.1×10^{-2}	1.48	5.9×10^{-2}
P06909	Complement component factor h (Cfh)	3.04	2.5×10^{-3}	4.46	1.3×10^{-3}	1.07	9.7×10^{-1}
P46412	Glutathione peroxidase 3 (Gpx3)	2.89	9.4×10^{-4}	3.55	1.2×10^{-4}	1.08	1.7×10^{-1}
P13707	Glycerol-3-phosphate dehydrogenase 1 (soluble) (Gpd1)	2.87	2.4×10^{-4}	2.69	4.4×10^{-4}	0.96	6.7×10^{-1}
P10107	Annexin A1 (Anxa1)	2.78	6.7×10^{-4}	2.50	6.4×10^{-3}	0.90	4.2×10^{-1}
P97290	Serine (or cysteine) peptidase inhibitor, clade G, member 1 (Serping1)	2.55	6.0×10^{-3}	3.72	2.6×10^{-3}	0.74	3.7×10^{-1}
Q00623	Apolipoprotein A-I (Apoa1)	2.41	2.6×10^{-3}	2.72	2.6×10^{-3}	0.72	1.7×10^{-1}
Q06890	Clusterin (Clu)	2.23	1.3×10^{-3}	3.05	1.6×10^{-3}	0.84	1.8×10^{-1}
P32848	Parvalbumin (Pvalb)	2.05	4.1×10^{-3}	2.60	3.1×10^{-2}	1.22	7.9×10^{-2}
Q91WP6	Serine (or cysteine) peptidase inhibitor, clade A, member 3N (Serpina3n)	2.05	2.0×10^{-3}	1.76	2.3×10^{-2}	1.18	2.7×10^{-2}

P07901	Heat shock protein 90, alpha (cytosolic), class A member 1 (Hsp90aa1)	2.03	3.0×10^{-3}	2.56	1.4×10^{-2}	1.06	6.0×10^{-1}
P04186	Complement factor B (Cfb)	1.98	2.8×10^{-3}	2.22	2.8×10^{-3}	0.97	8.1×10^{-1}
Q9ESB3	Histidine-rich glycoprotein (Hrg)	1.95	7.6×10^{-3}	2.89	1.1×10^{-2}	1.02	8.2×10^{-1}
Q9DBF1	Aldehyde dehydrogenase family 7, member A1 (Aldh7a1)	1.86	1.4×10^{-4}	1.63	1.4×10^{-3}	1.19	1.9×10^{-1}
Q9JII6	Aldo-keto reductase family 1, member A1 (aldehyde reductase) (Akr1a1)	1.86	1.8×10^{-4}	1.96	1.7×10^{-1}	1.20	6.3×10^{-1}
P19157	Glutathione S-transferase, pi 1 (Gstp1)	1.82	9.9×10^{-4}	2.50	1.4×10^{-3}	1.39	4.9×10^{-2}
Q791V5	Mitochondrial carrier 2 (Mtch2)	1.82	8.3×10^{-3}	1.68	1.1×10^{-1}	1.49	2.0×10^{-2}
P05064	Aldolase A, fructose-bisphosphate (Aldoa)	1.78	1.5×10^{-4}	1.53	4.8×10^{-3}	0.96	5.1×10^{-1}
P06745	Glucose phosphate isomerase 1 (Gpi1)	1.76	2.8×10^{-3}	1.80	3.8×10^{-3}	1.09	3.5×10^{-1}
P48758	Carbonyl reductase 1 (Cbr1)	1.68	8.4×10^{-4}	1.73	4.3×10^{-3}	1.40	6.2×10^{-3}
P13020	Gelsolin (Gsn)	1.66	2.3×10^{-3}	1.87	1.6×10^{-3}	0.96	7.2×10^{-1}
Q9WUB3	Muscle glycogen phosphorylase (Pygm)	1.64	2.9×10^{-3}	1.39	2.2×10^{-2}	0.89	1.3×10^{-1}
P54818	Galactosylceramidase (Galc)	1.62	8.5×10^{-3}	1.03	7.4×10^{-1}	1.12	1.0×10^{-2}
P09411	Phosphoglycerate kinase 1 (Pgk1)	1.62	8.8×10^{-4}	1.69	7.9×10^{-3}	1.18	5.8×10^{-3}
P14602	Heat shock protein 1 (Hspb1)	1.59	1.3×10^{-3}	1.80	2.7×10^{-3}	1.13	1.1×10^{-1}
P17563	Selenium binding protein 1 (Selenbp1)	1.57	4.1×10^{-5}	2.18	6.4×10^{-4}	1.47	2.4×10^{-2}
P45376	Aldo-keto reductase family 1, member B3 (aldose reductase) (Akr1b3)	1.56	7.4×10^{-3}	1.52	2.7×10^{-2}	1.10	4.2×10^{-1}
P62983	Ribosomal protein S27A (Rps27a)	1.55	4.5×10^{-3}	1.88	9.8×10^{-4}	1.01	8.5×10^{-1}
P16858	Glyceraldehyde-3-phosphate dehydrogenase (Gapdh)	1.53	6.8×10^{-3}	1.53	3.7×10^{-2}	1.04	8.2×10^{-1}
P09528	Ferritin heavy polypeptide 1 (Fth1)	1.51	2.3×10^{-3}	1.95	7.8×10^{-4}	1.25	4.5×10^{-1}
Q8R1G2	Carboxymethylenebutenolidase-like (Pseudomonas) (Cmbl)	1.48	7.0×10^{-3}	1.28	1.0×10^{-2}	1.07	4.9×10^{-1}

P24549	Aldehyde dehydrogenase family 1, subfamily A1 (Aldh1a1)	1.47	8.0×10^{-4}	1.43	4.3×10^{-3}	0.93	2.7×10^{-1}
P52480	Pyruvate kinase, muscle (Pkm)	1.46	2.5×10^{-3}	1.37	1.4×10^{-2}	1.05	4.2×10^{-1}
Q64105	Sepiapterin reductase (Spr)	1.45	5.2×10^{-3}	1.44	1.4×10^{-3}	1.18	1.8×10^{-1}
P47791	Glutathione reductase (Gsr)	1.43	4.1×10^{-3}	1.38	5.7×10^{-3}	1.04	8.9×10^{-1}
P51885	Lumican (Lum)	1.41	1.3×10^{-3}	1.51	3.9×10^{-3}	1.05	6.5×10^{-1}
P45377	Aldo-keto reductase family 1, member B8 (Akr1b8)	1.35	1.4×10^{-3}	1.42	3.2×10^{-2}	1.11	3.0×10^{-1}
Q99JB8	Protein kinase C and casein kinase substrate in neurons 3 (Pacsin3)	1.34	1.1×10^{-4}	1.46	1.1×10^{-4}	1.07	1.1×10^{-1}
Q9R0Y5	Adenylate kinase 1 (Ak1)	1.29	1.4×10^{-3}	1.31	8.7×10^{-3}	0.98	6.7×10^{-1}
Q60854	Serine (or cysteine) peptidase inhibitor, clade B, member 6a (Serpinb6a)	1.28	7.7×10^{-3}	1.35	3.3×10^{-4}	0.98	4.3×10^{-1}
Q9Z130	Heterogeneous nuclear ribonucleoprotein D-like (Hnrnpdl)	1.28	9.3×10^{-3}	1.31	1.6×10^{-1}	1.03	9.0×10^{-1}
P06151	Lactate dehydrogenase A (Ldha)	1.28	4.4×10^{-3}	1.12	7.6×10^{-2}	0.90	8.6×10^{-2}
Q8CDN6	Thioredoxin-like 1 (Txnl1)	1.22	8.3×10^{-3}	1.11	1.4×10^{-2}	1.11	9.8×10^{-3}
P47857	Phosphofructokinase, muscle (Pfkm)	1.21	3.8×10^{-3}	1.02	6.8×10^{-1}	0.90	6.4×10^{-2}
P62908	Ribosomal protein S3 (Rps3)	1.14	2.3×10^{-3}	0.88	3.4×10^{-1}	0.90	8.7×10^{-2}
Q3ULJ0	Glycerol-3-phosphate dehydrogenase 1-like (Gpd1l)	1.14	2.8×10^{-3}	1.19	1.4×10^{-4}	1.00	9.9×10^{-1}
P24527	Leukotriene A4 hydrolase (Lta4h)	1.14	8.3×10^{-4}	1.24	9.7×10^{-4}	0.97	4.2×10^{-1}
P63028	Tumor protein, translationally-controlled 1 (Tpt1)	1.14	1.0×10^{-3}	1.29	4.4×10^{-3}	1.04	5.5×10^{-1}
Q91Z53	Glyoxylate reductase/hydroxypyruvate reductase (Grhpr)	1.13	9.2×10^{-3}	1.18	7.4×10^{-3}	0.98	6.4×10^{-1}
Q9QYG0	N-myc downstream regulated gene 2 (Ndrg2)	1.12	3.9×10^{-3}	1.17	4.9×10^{-3}	1.03	4.8×10^{-1}
P28474	Alcohol dehydrogenase 5 (class III), chi polypeptide (Adh5)	1.08	2.5×10^{-3}	1.15	1.3×10^{-2}	0.99	7.9×10^{-1}
P01868	Immunoglobulin heavy constant gamma 1 (G1m marker) (Ighg1)	5.87	1.7×10^{-2}	6.29	6.1×10^{-4}	2.33	1.7×10^{-1}

Q8VCM7	Fibrinogen gamma chain (Fgg)	3.81	1.1×10^{-2}	4.08	8.6×10^{-3}	0.80	6.3×10^{-1}
Q80YC5	Coagulation factor XII (Hageman factor) (F12)	2.09	1.1×10^{-2}	3.67	6.8×10^{-4}	0.79	3.0×10^{-1}
P70695	Fructose bisphosphatase 2 (Fbp2)	2.46	1.5×10^{-2}	2.71	2.4×10^{-3}	1.16	3.4×10^{-1}
P32261	Serine (or cysteine) peptidase inhibitor, clade C (antithrombin), member 1 (Serpinc1)	2.49	1.1×10^{-2}	2.46	5.1×10^{-3}	0.77	2.9×10^{-1}
Q9DAK9	Phosphohistidine phosphatase 1 (Phpt1)	1.49	1.1×10^{-2}	1.77	1.5×10^{-3}	1.17	9.5×10^{-2}
Q9CRB6	Tubulin polymerization-promoting protein family member 3 (Tppp3)	1.53	9.4×10^{-2}	1.76	8.4×10^{-3}	1.55	1.4×10^{-2}
O88668	Cellular repressor of E1A-stimulated genes 1 (Creg1)	1.44	2.9×10^{-2}	1.73	6.3×10^{-3}	1.42	7.6×10^{-2}
P11352	Glutathione peroxidase 1 (Gpx1)	1.26	5.0×10^{-2}	1.55	6.1×10^{-3}	1.03	6.4×10^{-1}
Q60847	Collagen, type XII, alpha 1 (Col12a1)	1.36	1.5×10^{-2}	1.53	1.1×10^{-3}	1.20	1.5×10^{-1}
P48774	Glutathione S-transferase, mu 5 (Gstm5)	1.20	1.4×10^{-2}	1.48	6.2×10^{-4}	0.99	8.1×10^{-1}
P53994	RAB2A, member RAS oncogene family (Rab2a)	1.20	3.6×10^{-2}	1.41	1.0×10^{-3}	1.03	6.9×10^{-1}
O88844	Isocitrate dehydrogenase 1 (NADP+), soluble (Idh1)	0.94	4.6×10^{-1}	1.41	1.7×10^{-3}	1.00	9.2×10^{-1}
P40142	Transketolase (Tkt)	1.24	5.9×10^{-2}	1.41	2.4×10^{-3}	1.09	5.2×10^{-1}
P47199	Crystallin, zeta (Cryz)	1.20	1.0×10^{-2}	1.35	6.2×10^{-4}	1.02	8.4×10^{-1}
Q9JHW2	Nitrilase family, member 2 (Nit2)	1.05	6.6×10^{-1}	1.34	5.4×10^{-4}	1.05	1.7×10^{-1}
Q9JK53	Proline arginine-rich end leucine-rich repeat (Prelp)	1.07	2.9×10^{-1}	1.33	5.7×10^{-3}	1.06	3.1×10^{-1}
Q8BVI4	Quinoid dihydropteridine reductase (Qdpr)	1.26	4.8×10^{-2}	1.32	3.6×10^{-3}	1.05	6.8×10^{-1}
Q99LX0	Parkinson disease (autosomal recessive, early onset) 7 (Park7)	1.26	2.1×10^{-2}	1.31	7.4×10^{-3}	0.99	7.7×10^{-1}
P16045	Lectin, galactose binding, soluble 1 (Lgals1)	1.15	1.8×10^{-2}	1.29	4.2×10^{-4}	1.02	7.5×10^{-1}

P63005	Platelet-activating factor acetylhydrolase, isoform 1b, subunit 1 (Pafah1b1)	1.16	1.8×10^{-2}	1.29	3.7×10^{-3}	1.15	5.4×10^{-2}
P97384	Annexin A11 (Anxa11)	1.13	3.9×10^{-2}	1.26	2.7×10^{-3}	1.04	3.1×10^{-1}
P09405	Nucleolin (Ncl)	1.02	6.4×10^{-1}	1.25	9.2×10^{-4}	1.07	5.9×10^{-1}
Q99PT1	Rho GDP dissociation inhibitor (GDI) alpha (Arhgdia)	1.07	3.0×10^{-2}	1.17	4.2×10^{-3}	1.08	7.0×10^{-2}
Q02053	Ubiquitin-like modifier activating enzyme 1 (Uba1)	1.06	7.0×10^{-2}	1.16	1.2×10^{-3}	1.02	5.6×10^{-1}
Q9CQ60	6-phosphogluconolactonase (Pgls)	0.92	4.2×10^{-1}	1.12	5.4×10^{-3}	1.01	8.3×10^{-1}
P62071	Related RAS viral (r-ras) oncogene 2 (Rras2)	1.03	7.8×10^{-1}	1.35	9.1×10^{-1}	1.74	4.5×10^{-4}
O35215	D-dopachrome tautomerase (Ddt)	1.03	9.4×10^{-1}	1.46	7.4×10^{-2}	1.67	7.3×10^{-3}
P70349	Histidine triad nucleotide binding protein 1 (Hint1)	1.43	1.5×10^{-2}	1.95	1.2×10^{-1}	1.45	5.3×10^{-3}
Q8K183	Pyridoxal (pyridoxine, vitamin B6) kinase (Pdxk)	1.28	3.3×10^{-2}	1.13	2.0×10^{-1}	1.41	4.8×10^{-3}
O09131	Glutathione S-transferase omega 1 (Gsto1)	0.87	1.4×10^{-1}	0.97	6.3×10^{-1}	1.26	6.3×10^{-3}
P62814	ATPase, H+ transporting, lysosomal V1 subunit B2 (Atp6v1b2)	1.03	4.0×10^{-1}	1.10	3.9×10^{-2}	1.18	6.1×10^{-3}
Q6PIE5	ATPase, Na+/K+ transporting, alpha 2 polypeptide (Atp1a2)	1.15	5.0×10^{-2}	1.11	3.9×10^{-2}	1.18	5.4×10^{-3}
Q9QUI0	Ras homolog family member A (Rhoa)	0.94	2.0×10^{-1}	1.05	4.7×10^{-1}	1.09	6.0×10^{-3}
P97355	Spermine synthase (Sms)	0.13	4.7×10^{-3}	2.09	9.4×10^{-1}	0.52	2.1×10^{-1}
O88746	Target of myb1 trafficking protein (Tom1)	0.35	4.2×10^{-4}	0.43	5.3×10^{-4}	1.05	5.8×10^{-1}
O35459	Enoyl coenzyme A hydratase 1, peroxisomal (Ech1)	0.38	6.1×10^{-4}	0.46	1.1×10^{-3}	0.92	3.7×10^{-1}
Q9R0Q7	Prostaglandin E synthase 3 (cytosolic) (Ptges3)	0.40	5.1×10^{-3}	0.25	3.1×10^{-2}	1.15	5.4×10^{-1}
Q8CI43	Myosin, light polypeptide 6B (Myl6b)	0.43	6.3×10^{-3}	0.47	3.6×10^{-3}	1.44	9.7×10^{-2}

Q9CYH2	Family with sequence similarity 213, member A (Fam213a)	0.46	3.0×10^{-4}	0.73	4.1×10^{-2}	1.09	5.0×10^{-1}
P34914	Epoxide hydrolase 2, cytoplasmic (Ephx2)	0.48	5.4×10^{-4}	0.60	9.9×10^{-4}	1.08	3.0×10^{-1}
P23927	Crystallin, alpha B (Cryab)	0.48	1.1×10^{-3}	0.54	1.7×10^{-5}	1.10	8.7×10^{-2}
P24270	Catalase (Cat)	0.49	3.5×10^{-3}	0.62	1.0×10^{-2}	1.12	5.6×10^{-1}
Q8VDN2	ATPase, Na+/K+ transporting, alpha 1 polypeptide (Atp1a1)	0.49	6.7×10^{-3}	0.55	1.6×10^{-2}	1.13	2.3×10^{-1}
P31001	Desmin (Des)	0.52	2.6×10^{-4}	0.51	2.6×10^{-4}	1.07	2.9×10^{-1}
Q04447	Creatine kinase, brain (Ckb)	0.52	5.5×10^{-3}	1.06	8.3×10^{-1}	1.34	2.6×10^{-4}
P45952	Acyl-Coenzyme A dehydrogenase, medium chain (Acadm)	0.53	1.5×10^{-3}	0.61	2.8×10^{-4}	0.94	2.3×10^{-1}
P99024	Tubulin, beta 5 class I (Tubb5)	0.53	2.2×10^{-5}	0.59	1.5×10^{-2}	0.98	7.8×10^{-1}
Q99LB2	Dehydrogenase/reductase (SDR family) member 4 (Dhrs4)	0.55	1.6×10^{-3}	0.69	1.4×10^{-2}	0.85	7.1×10^{-2}
P54071	Isocitrate dehydrogenase 2 (NADP+), mitochondrial (Idh2)	0.57	4.4×10^{-4}	0.70	3.9×10^{-3}	1.04	6.6×10^{-1}
P11404	Fatty acid binding protein 3, muscle and heart (Fabp3)	0.57	9.4×10^{-3}	0.77	2.1×10^{-2}	1.03	8.4×10^{-1}
P62082	Ribosomal protein S7 (Rps7)	0.58	7.4×10^{-3}	0.77	2.3×10^{-1}	0.99	9.1×10^{-1}
P26041	Moesin (Msn)	0.58	7.2×10^{-3}	0.76	9.4×10^{-2}	0.95	3.4×10^{-1}
P20152	Vimentin (Vim)	0.59	3.0×10^{-3}	0.65	2.0×10^{-3}	0.90	4.8×10^{-1}
Q99KK7	Dipeptidylpeptidase 3 (Dpp3)	0.60	2.1×10^{-3}	0.75	1.2×10^{-2}	1.01	9.3×10^{-1}
P62830	Ribosomal protein L23 (Rpl23)	0.61	1.0×10^{-4}	0.74	1.8×10^{-1}	1.05	4.4×10^{-1}
Q9D8W5	Proteasome (prosome, macropain) 26S subunit, non-ATPase, 12 (Psmd12)	0.61	9.5×10^{-3}	0.89	4.8×10^{-1}	0.84	3.7×10^{-1}
P15532	NME/NM23 nucleoside diphosphate kinase 1 (Nme1)	0.62	8.3×10^{-3}	0.84	2.5×10^{-1}	0.87	4.1×10^{-1}
P62196	Protease (prosome, macropain) 26S subunit, ATPase 5 (Psmc5)	0.62	1.6×10^{-3}	0.78	2.6×10^{-1}	0.92	5.5×10^{-1}
Q9EQH3	VPS35 retromer complex component (Vps35)	0.62	6.2×10^{-3}	0.72	4.4×10^{-2}	1.01	9.6×10^{-1}

Q8BWT1	Acetyl-Coenzyme A acyltransferase 2 (mitochondrial 3-oxoacyl-Coenzyme A thiolase) (Acaa2)	0.63	1.8×10^{-3}	0.71	1.0×10^{-3}	0.95	2.7×10^{-1}
P42125	Enoyl-Coenzyme A delta isomerase 1 (Eci1)	0.64	1.8×10^{-3}	0.72	1.1×10^{-3}	0.93	3.4×10^{-1}
Q6GSS7	Histone cluster 2, H2aa1 (Hist2h2aa1)	0.65	3.1×10^{-3}	0.93	5.5×10^{-1}	1.03	6.9×10^{-1}
Q9QYR9	Acyl-CoA thioesterase 2 (Acot2)	0.65	1.4×10^{-3}	0.75	6.2×10^{-3}	0.98	8.2×10^{-1}
Q99JY0	Hydroxyacyl-Coenzyme A dehydrogenase/3-ketoacyl-Coenzyme A thiolase/enoyl-Coenzyme A hydratase (trifunctional protein), beta subunit (Hadhb)	0.65	9.9×10^{-5}	0.78	1.3×10^{-2}	0.95	4.5×10^{-1}
Q91ZJ5	UDP-glucose pyrophosphorylase 2 (Ugp2)	0.66	6.0×10^{-4}	0.69	2.6×10^{-3}	0.95	2.0×10^{-1}
P50518	ATPase, H+ transporting, lysosomal V1 subunit E1 (Atp6v1e1)	0.67	3.2×10^{-3}	0.86	9.5×10^{-2}	1.09	3.0×10^{-1}
Q9CQ62	2,4-dienoyl CoA reductase 1, mitochondrial (Decr1)	0.68	1.3×10^{-3}	0.77	6.0×10^{-3}	0.98	7.3×10^{-1}
P21107	Tropomyosin 3, gamma (Tpm3)	0.68	8.8×10^{-3}	0.71	1.7×10^{-2}	1.14	2.1×10^{-1}
Q6ZWY9	Histone cluster 1, H2bc (Hist1h2bc)	0.68	9.6×10^{-3}	0.97	7.5×10^{-1}	1.01	9.3×10^{-1}
Q8QZT1	Acetyl-Coenzyme A acetyltransferase 1 (Acat1)	0.68	2.9×10^{-4}	0.75	2.2×10^{-3}	0.93	8.3×10^{-2}
P00405	Cytochrome c oxidase subunit II (COX2)	0.69	6.9×10^{-3}	0.78	3.0×10^{-2}	0.84	1.0×10^{-1}
P18242	Cathepsin D (Ctsd)	0.70	4.9×10^{-3}	0.96	5.8×10^{-1}	0.99	8.7×10^{-1}
Q9JM76	Actin related protein 2/3 complex, subunit 3 (Arpc3)	0.70	2.9×10^{-3}	0.82	2.0×10^{-3}	1.03	6.4×10^{-1}
Q9DBL1	Acyl-Coenzyme A dehydrogenase, short/branched chain (Acadsb)	0.70	5.9×10^{-3}	0.83	6.4×10^{-2}	0.90	2.8×10^{-1}
P60867	Ribosomal protein S20 (Rps20)	0.71	2.7×10^{-3}	0.84	1.7×10^{-2}	0.87	3.8×10^{-2}
Q99LC5	Electron transferring flavoprotein, alpha polypeptide (Etfa)	0.72	3.0×10^{-3}	0.88	5.1×10^{-2}	0.95	3.3×10^{-1}
Q9CQN1	TNF receptor-associated protein 1 (Trap1)	0.72	9.8×10^{-3}	0.90	4.2×10^{-1}	0.96	5.8×10^{-1}

Q99KQ4	Nicotinamide phosphoribosyltransferase (Nampt)	0.72	2.9×10^{-3}	0.79	1.1×10^{-2}	0.96	6.4×10^{-1}
P68037	Ubiquitin-conjugating enzyme E2L 3 (Ube2l3)	0.73	7.1×10^{-3}	0.86	2.0×10^{-1}	0.96	6.3×10^{-1}
Q62425	NADH dehydrogenase (ubiquinone) 1 alpha subcomplex, 4 (Ndufa4)	0.73	7.4×10^{-3}	0.82	5.7×10^{-2}	0.94	4.4×10^{-1}
Q8QZS1	3-hydroxyisobutyryl-Coenzyme A hydrolase (Hibch)	0.74	2.2×10^{-3}	0.87	2.2×10^{-2}	0.94	5.4×10^{-2}
P27773	Protein disulfide isomerase associated 3 (Pdia3)	0.74	5.5×10^{-3}	0.75	1.7×10^{-3}	0.87	5.7×10^{-2}
P38647	Heat shock protein 9 (Hspa9)	0.74	2.7×10^{-3}	0.76	5.6×10^{-3}	0.95	4.3×10^{-1}
P16125	Lactate dehydrogenase B (Ldhb)	0.74	6.7×10^{-3}	0.82	3.3×10^{-3}	1.05	5.1×10^{-1}
Q9D2G2	Dihydrolipoamide S-succinyltransferase (E2 component of 2-oxo-glutarate complex) (Dlst)	0.75	3.8×10^{-3}	0.81	7.4×10^{-3}	0.90	8.6×10^{-2}
Q8VDQ1	Prostaglandin reductase 2 (Ptgr2)	0.75	7.9×10^{-3}	0.84	1.9×10^{-1}	1.13	1.3×10^{-1}
Q9EQP2	EH-domain containing 4 (Ehd4)	0.75	2.1×10^{-3}	0.89	1.5×10^{-1}	0.99	8.5×10^{-1}
Q99M71	Ependymin related protein 1 (zebrafish) (Epdr1)	0.75	5.1×10^{-3}	0.75	3.5×10^{-3}	1.12	6.1×10^{-1}
Q9CQM5	Thioredoxin domain containing 17 (Txndc17)	0.76	6.4×10^{-3}	1.21	2.9×10^{-1}	0.94	6.3×10^{-1}
Q9CPY7	Leucine aminopeptidase 3 (Lap3)	0.76	3.9×10^{-3}	0.84	3.2×10^{-2}	1.03	7.4×10^{-1}
Q64010	V-crk avian sarcoma virus CT10 oncogene homolog (Crk)	0.76	9.0×10^{-3}	0.87	2.3×10^{-1}	0.87	7.4×10^{-2}
P26516	Proteasome (prosome, macropain) 26S subunit, non-ATPase, 7 (Psmd7)	0.76	6.9×10^{-3}	0.76	1.2×10^{-2}	1.04	7.7×10^{-1}
Q8VEM8	Solute carrier family 25 (mitochondrial carrier, phosphate carrier), member 3 (Slc25a3)	0.76	8.0×10^{-3}	0.83	1.4×10^{-2}	0.99	9.1×10^{-1}
P12787	Cytochrome c oxidase subunit Va (Cox5a)	0.76	7.7×10^{-3}	0.87	1.6×10^{-1}	1.03	7.8×10^{-1}
P08113	Heat shock protein 90, beta (Grp94), member 1 (Hsp90b1)	0.77	7.1×10^{-3}	0.78	1.1×10^{-1}	1.03	7.1×10^{-1}

Q9DCX2	ATP synthase, H+ transporting, mitochondrial F0 complex, subunit D (Atp5h)	0.77	6.9×10^{-3}	0.87	1.7×10^{-2}	0.96	4.9×10^{-1}
Q9DCM2	Glutathione S-transferase kappa 1 (Gstk1)	0.77	1.5×10^{-4}	1.02	8.0×10^{-1}	0.99	8.3×10^{-1}
P19783	Cytochrome c oxidase subunit IV isoform 1 (Cox4i1)	0.78	1.6×10^{-3}	0.80	4.0×10^{-3}	0.91	1.4×10^{-1}
P62821	RAB1A, member RAS oncogene family (Rab1a)	0.78	3.3×10^{-3}	0.70	4.5×10^{-2}	1.05	6.4×10^{-1}
P48771	Cytochrome c oxidase subunit VIIa 2 (Cox7a2)	0.79	2.4×10^{-3}	0.84	8.1×10^{-2}	0.92	2.9×10^{-1}
Q9CZU6	Citrate synthase (Cs)	0.79	6.5×10^{-4}	0.87	2.7×10^{-3}	0.93	5.4×10^{-2}
Q9QXS1	Plectin (Plec)	0.80	2.3×10^{-3}	0.81	2.0×10^{-3}	1.03	1.6×10^{-1}
P97807	Fumarate hydratase 1 (Fh1)	0.81	5.9×10^{-3}	1.00	1.0	0.88	4.8×10^{-2}
Q9CQM9	Glutaredoxin 3 (Glrx3)	0.82	2.6×10^{-3}	0.94	2.5×10^{-1}	0.91	2.1×10^{-1}
Q9D051	Pyruvate dehydrogenase (lipoamide) beta (Pdhb)	0.82	5.2×10^{-3}	0.91	5.5×10^{-2}	0.92	2.3×10^{-1}
Q9JI91	Actinin alpha 2 (Actn2)	0.82	1.1×10^{-3}	0.88	9.6×10^{-4}	1.09	7.5×10^{-2}
P08249	Malate dehydrogenase 2, NAD (mitochondrial) (Mdh2)	0.82	4.1×10^{-3}	0.91	1.6×10^{-1}	0.91	6.4×10^{-2}
Q8R164	Biphenyl hydrolase-like (serine hydrolase, breast epithelial mucin-associated antigen) (Bphl)	0.83	8.8×10^{-3}	0.92	1.3×10^{-1}	0.93	6.1×10^{-2}
Q9DB77	Ubiquinol cytochrome c reductase core protein 2 (Uqcrc2)	0.83	2.5×10^{-4}	0.91	1.7×10^{-1}	0.89	8.6×10^{-2}
P32921	Tryptophanyl-tRNA synthetase (Wars)	0.84	5.9×10^{-3}	0.94	1.9×10^{-1}	1.00	9.7×10^{-1}
P61027	RAB10, member RAS oncogene family (Rab10)	0.85	8.1×10^{-3}	0.84	1.3×10^{-1}	0.95	3.8×10^{-1}
P47738	Aldehyde dehydrogenase 2, mitochondrial (Aldh2)	0.85	9.5×10^{-3}	0.95	1.8×10^{-1}	0.98	7.0×10^{-1}
Q9D6Y9	Glucan (1,4-alpha-), branching enzyme 1 (Gbe1)	0.86	5.6×10^{-3}	0.97	6.2×10^{-1}	1.03	4.8×10^{-1}

P05202	Glutamatic-oxaloacetic transaminase 2, mitochondrial (Got2)	0.86	1.6×10^{-3}	0.94	2.6×10^{-1}	0.96	3.8×10^{-1}
P62141	Protein phosphatase 1, catalytic subunit, beta isoform (Ppp1cb)	0.87	4.0×10^{-3}	0.94	1.4×10^{-1}	1.04	3.6×10^{-1}
P26638	Seryl-aminoacyl-tRNA synthetase (Sars)	0.88	5.0×10^{-3}	0.81	1.8×10^{-1}	1.01	8.6×10^{-1}
Q61398	Procollagen C-endopeptidase enhancer protein (Pcolce)	0.24	6.1×10^{-2}	0.12	4.9×10^{-3}	0.95	7.4×10^{-1}
P43025	C-type lectin domain family 3, member b (Clec3b)	0.04	4.0×10^{-2}	0.23	3.7×10^{-3}	0.40	9.7×10^{-2}
P08030	Adenine phosphoribosyl transferase (Aprt)	0.51	3.2×10^{-2}	0.37	4.1×10^{-3}	1.22	3.0×10^{-1}
P62270	Ribosomal protein S18 (Rps18)	0.29	3.7×10^{-2}	0.40	5.6×10^{-3}	1.00	9.8×10^{-1}
Q9EQK5	Major vault protein (Mvp)	0.32	1.6×10^{-2}	0.40	6.6×10^{-3}	1.18	2.8×10^{-1}
P27546	Microtubule-associated protein 4 (Map4)	0.53	3.5×10^{-2}	0.46	2.3×10^{-3}	1.12	3.6×10^{-1}
O88587	Catechol-O-methyltransferase (Comt)	0.63	1.7×10^{-2}	0.50	9.2×10^{-3}	1.31	1.0×10^{-1}
Q9R0P9	Ubiquitin carboxy-terminal hydrolase L1 (Uchl1)	0.59	2.4×10^{-2}	0.52	6.0×10^{-3}	1.21	1.8×10^{-1}
Q99L47	Suppression of tumorigenicity 13 (St13)	0.57	1.6×10^{-2}	0.53	5.0×10^{-4}	1.10	1.7×10^{-1}
P97298	Serine (or cysteine) peptidase inhibitor, clade F, member 1 (Serpinf1)	0.51	4.3×10^{-2}	0.53	2.3×10^{-3}	0.86	4.6×10^{-1}
Q9D5V6	Synapse associated protein 1 (Syap1)	0.76	2.6×10^{-2}	0.62	1.9×10^{-3}	1.06	5.3×10^{-2}
Q9CPV4	Glyoxalase domain containing 4 (Glod4)	0.61	1.6×10^{-2}	0.65	2.9×10^{-3}	1.05	6.7×10^{-1}
Q9WVA4	Transgelin 2 (Tagln2)	0.57	1.8×10^{-2}	0.73	4.1×10^{-3}	0.88	1.6×10^{-1}
Q8C1B7	Septin 11 (Sept11)	0.75	1.3×10^{-2}	0.75	2.4×10^{-4}	1.00	9.8×10^{-1}
Q922B2	Aspartyl-tRNA synthetase (Dars)	0.92	4.3×10^{-1}	0.78	4.3×10^{-3}	0.95	5.0×10^{-1}

P97372	Proteasome (prosome, macropain) activator subunit 2 (PA28 beta) (Psme2)	0.91	2.0×10^{-1}	0.79	4.8×10^{-3}	1.12	2.9×10^{-1}
Q9CVB6	Actin related protein 2/3 complex, subunit 2 (Arpc2)	0.70	1.3×10^{-2}	0.80	4.1×10^{-3}	0.96	6.7×10^{-1}
O35593	Proteasome (prosome, macropain) 26S subunit, non-ATPase, 14 (Psmd14)	0.85	1.4×10^{-1}	0.81	5.9×10^{-3}	1.18	4.1×10^{-2}
Q11011	Aminopeptidase puromycin sensitive (Npepps)	0.78	1.0×10^{-2}	0.83	7.8×10^{-3}	1.00	9.4×10^{-1}

b: The proteins in EDL muscles

**Accession**	**Gene name**	**μ-*g* & FOS (−) /A1-*g* & FOS (−)**		**μ-*g* & FOS (+) /A1-*g* & FOS (−)**		**A1-*g* & FOS (+) /A1-*g* & FOS (−)**	
		**Fold change**	***P* value**	**Fold change**	***P* value**	**Fold change**	***P* value**
P54071	Isocitrate dehydrogenase 2 (NADP+), mitochondrial (Idh2)	1.43	8.5×10^{-4}	1.45	3.2×10^{-4}	0.98	6.3×10^{-1}
P04247	Myoglobin (Mb)	1.38	1.1×10^{-3}	1.57	2.2×10^{-3}	0.98	7.6×10^{-1}
P16125	Lactate dehydrogenase B (Ldhb)	1.35	2.1×10^{-3}	1.41	3.2×10^{-3}	0.95	5.2×10^{-1}
P01027	Complement component 3 (C3)	1.35	8.3×10^{-3}	1.48	4.5×10^{-2}	1.17	1.1×10^{-1}
P14094	ATPase, Na+/K+ transporting, beta 1 polypeptide (Atp1b1)	1.32	2.5×10^{-4}	1.48	1.1×10^{-3}	1.10	1.8×10^{-1}
O09131	Glutathione S-transferase omega 1 (Gsto1)	1.30	2.2×10^{-3}	1.41	1.6×10^{-4}	1.00	9.8×10^{-1}
P47791	Glutathione reductase (Gsr)	1.21	2.6×10^{-3}	1.30	1.1×10^{-2}	0.92	2.0×10^{-1}
P28271	Aconitase 1 (Aco1)	1.21	5.9×10^{-3}	1.21	8.0×10^{-2}	0.96	3.7×10^{-1}
Q8CDN6	Thioredoxin-like 1 (Txnl1)	1.19	9.8×10^{-3}	1.14	5.0×10^{-2}	1.07	1.3×10^{-1}
O88342	WD repeat domain 1 (Wdr1)	1.18	5.3×10^{-3}	1.23	9.4×10^{-4}	0.99	8.3×10^{-1}
P47199	Crystallin, zeta (Cryz)	1.12	7.1×10^{-3}	1.11	1.3×10^{-2}	1.06	1.2×10^{-1}
Q11011	Aminopeptidase puromycin sensitive (Npepps)	1.08	5.9×10^{-3}	1.05	2.3×10^{-1}	0.98	4.2×10^{-1}
Q9DBB9	Carboxypeptidase N, polypeptide 2 (Cpn2)	1.00	1.0	Infinity	3.4×10^{-5}	Infinity (High)	3.7×10^{-1}
P13634	Carbonic anhydrase 1 (Car1)	1.27	1.9×10^{-1}	1.86	4.4×10^{-3}	1.13	7.0×10^{-1}

Q8VD04	GRIP1 associated protein 1 (Gripap1)	1.38	3.3×10^{-1}	1.78	2.2×10^{-3}	1.15	2.1×10^{-1}
Q5SUV5	Myosin light chain kinase family, member 4 (Mylk4)	1.35	1.3×10^{-2}	1.65	1.3×10^{-3}	0.98	8.0×10^{-1}
P07309	Transthyretin (Ttr)	1.15	3.0×10^{-1}	1.47	6.9×10^{-3}	0.95	5.6×10^{-1}
P22599	Serine (or cysteine) preptidase inhibitor, clade A, member 1B (Serpina1b)	1.17	1.3×10^{-1}	1.43	5.6×10^{-3}	1.03	8.4×10^{-1}
P07724	Albumin (Alb)	1.13	2.5×10^{-1}	1.40	7.2×10^{-3}	1.03	7.7×10^{-1}
P00920	Carbonic anhydrase 2 (Car2)	1.08	2.0×10^{-1}	1.37	7.1×10^{-3}	1.02	9.6×10^{-1}
P07759	Serine (or cysteine) peptidase inhibitor, clade A, member 3K (Serpina3k)	1.24	1.6×10^{-1}	1.36	5.3×10^{-3}	0.93	4.7×10^{-1}
Q6P1B1	X-prolyl aminopeptidase (aminopeptidase P) 1, soluble (Xpnpep1)	1.30	3.3×10^{-2}	1.32	4.8×10^{-3}	1.03	7.6×10^{-1}
Q9D6Y9	Glucan (1,4-alpha-), branching enzyme 1 (Gbe1)	1.22	1.6×10^{-2}	1.29	8.6×10^{-4}	0.96	5.3×10^{-1}
Q921I1	Transferrin (Trf)	1.11	3.6×10^{-1}	1.29	4.0×10^{-3}	0.92	3.4×10^{-1}
Q9JI91	Actinin alpha 2 (Actn2)	1.17	3.5×10^{-2}	1.28	6.2×10^{-3}	1.04	4.8×10^{-1}
Q9D358	Acid phosphatase 1, soluble (Acp1)	1.12	2.3×10^{-2}	1.25	3.6×10^{-3}	1.01	9.4×10^{-1}
Q91VF2	Histamine N-methyltransferase (Hnmt)	1.01	9.1×10^{-1}	1.25	5.7×10^{-3}	1.17	3.5×10^{-1}
Q9DCW4	Electron transferring flavoprotein, beta polypeptide (Etfb)	1.15	1.5×10^{-2}	1.19	3.5×10^{-3}	1.01	6.4×10^{-1}
P20108	Peroxiredoxin 3 (Prdx3)	1.04	4.5×10^{-1}	1.18	7.7×10^{-3}	1.04	5.2×10^{-1}
P31786	Diazepam binding inhibitor (Dbi)	1.10	7.6×10^{-2}	1.18	8.6×10^{-3}	1.07	2.4×10^{-1}
Q99LC5	Electron transferring flavoprotein, alpha polypeptide (Etfa)	1.13	1.1×10^{-2}	1.17	2.4×10^{-3}	1.00	9.6×10^{-1}
P99029	Peroxiredoxin 5 (Prdx5)	1.06	1.2×10^{-1}	1.13	1.0×10^{-4}	0.95	1.6×10^{-1}
Q9Z2Y8	Proline synthetase co-transcribed (Prosc)	1.10	1.3×10^{-2}	1.10	9.8×10^{-3}	1.04	4.8×10^{-1}
P14152	Malate dehydrogenase 1, NAD (soluble) (Mdh1)	1.05	1.4×10^{-1}	1.09	6.2×10^{-3}	0.99	8.0×10^{-1}

Q8BTM8	Filamin, alpha (Flna)	Infinity	4.8×10^{-5}	0.71	4.1×10^{-1}	1.04	4.9×10^{-1}
P14148	Ribosomal protein L7 (Rpl7)	0.57	2.1×10^{-3}	0.61	8.1×10^{-3}	0.76	8.1×10^{-2}
P62082	Ribosomal protein S7 (Rps7)	0.59	6.4×10^{-3}	0.55	4.0×10^{-3}	0.68	1.6×10^{-1}
Q9CXW4	Ribosomal protein L11 (Rpl11)	0.68	3.5×10^{-3}	0.74	1.7×10^{-2}	0.87	3.2×10^{-1}
P14602	Heat shock protein 1 (Hspb1)	0.70	4.9×10^{-3}	0.76	1.3×10^{-2}	1.17	3.7×10^{-1}
Q922B2	Aspartyl-tRNA synthetase (Dars)	0.71	7.9×10^{-3}	0.59	3.7×10^{-3}	0.85	1.0×10^{-1}
P70670	Nascent polypeptide-associated complex alpha polypeptide (Naca)	0.72	4.5×10^{-4}	0.66	3.7×10^{-4}	0.88	4.1×10^{-2}
P08113	Heat shock protein 90, beta (Grp94), member 1 (Hsp90b1)	0.73	2.5×10^{-3}	0.60	5.2×10^{-4}	0.82	7.4×10^{-2}
P60710	Actin, beta (Actb)	0.76	2.7×10^{-3}	0.72	2.0×10^{-2}	0.90	4.7×10^{-1}
P99027	Ribosomal protein, large P2 (Rplp2)	0.76	4.6×10^{-3}	0.76	1.0×10^{-2}	0.95	5.1×10^{-1}
Q9CQ65	Methylthioadenosine phosphorylase (Mtap)	0.79	4.2×10^{-3}	0.78	2.1×10^{-2}	1.19	1.5×10^{-2}
P11499	Heat shock protein 90 alpha (cytosolic), class B member 1 (Hsp90ab1)	0.80	6.5×10^{-3}	0.72	2.7×10^{-3}	0.93	2.2×10^{-1}
P68040	Receptor for activated C kinase 1 (Rack1)	0.81	8.6×10^{-3}	0.76	4.3×10^{-3}	0.90	1.2×10^{-1}
Q01853	valosin containing protein (Vcp)	0.81	6.8×10^{-3}	0.82	7.0×10^{-3}	1.00	9.7×10^{-1}
P14211	Calreticulin (Calr)	0.81	5.8×10^{-3}	0.79	4.1×10^{-3}	0.91	1.4×10^{-1}
P14206	Ribosomal protein SA (Rpsa)	0.81	6.5×10^{-3}	0.78	4.4×10^{-3}	0.90	1.2×10^{-1}
Q64105	Sepiapterin reductase (Spr)	0.82	2.3×10^{-3}	0.80	1.5×10^{-2}	1.00	9.5×10^{-1}
P34914	Epoxide hydrolase 2, cytoplasmic (Ephx2)	0.83	8.8×10^{-3}	0.85	8.7×10^{-2}	0.99	8.4×10^{-1}
P62141	Protein phosphatase 1, catalytic subunit, beta isoform (Ppp1cb)	0.85	3.9×10^{-3}	0.85	9.6×10^{-3}	0.95	2.8×10^{-1}
P14824	Annexin A6 (Anxa6)	0.86	2.9×10^{-3}	0.84	1.4×10^{-3}	0.98	5.1×10^{-1}
Q9CWJ9	5-aminoimidazole-4-carboxamide ribonucleotide formyltransferase/IMP cyclohydrolase (Atic)	0.90	5.6×10^{-3}	0.88	1.5×10^{-1}	1.01	1.0
Q8VED5	Keratin 79 (Krt79)	1.04	5.5×10^{-1}	0.06	2.5×10^{-3}	0.37	3.0×10^{-2}

P80318	Chaperonin containing Tcp1, subunit 3 (gamma) (Cct3)	0.61	1.3×10^{-2}	0.45	2.8×10^{-3}	0.84	2.4×10^{-1}
P47857	Phosphofructokinase, muscle (Pfkm)	0.78	2.4×10^{-2}	0.55	3.0×10^{-3}	0.92	4.1×10^{-1}
P80314	Chaperonin containing Tcp1, subunit 2 (beta) (Cct2)	0.69	3.2×10^{-2}	0.60	1.6×10^{-3}	0.85	6.0×10^{-2}
Q9D0I9	Arginyl-tRNA synthetase (Rars)	0.74	2.7×10^{-2}	0.61	3.1×10^{-3}	0.89	3.3×10^{-1}
Q8VCT3	Arginyl aminopeptidase (aminopeptidase B) (Rnpep)	0.88	2.1×10^{-2}	0.66	1.5×10^{-4}	1.01	9.7×10^{-1}
Q9D8N0	Eukaryotic translation elongation factor 1 gamma (Eef1g)	0.84	3.3×10^{-2}	0.69	1.7×10^{-3}	0.92	2.3×10^{-1}
P62274	Ribosomal protein S29 (Rps29)	0.79	3.4×10^{-2}	0.70	5.8×10^{-3}	0.98	8.0×10^{-1}
Q8K183	Pyridoxal (pyridoxine, vitamin B6) kinase (Pdxk)	0.88	1.3×10^{-1}	0.77	6.8×10^{-3}	0.95	4.8×10^{-1}
P68033	Actin, alpha, cardiac muscle 1 (Actc1)	0.99	9.0×10^{-1}	0.77	1.9×10^{-3}	0.95	6.1×10^{-1}
P58252	Eukaryotic translation elongation factor 2 (Eef2)	0.87	1.5×10^{-2}	0.80	7.2×10^{-4}	0.95	4.9×10^{-1}
P15327	2,3-bisphosphoglycerate mutase (Bpgm)	0.84	6.6×10^{-2}	0.84	6.9×10^{-4}	1.02	4.4×10^{-1}
Q93092	Transaldolase 1 (Taldo1)	0.90	1.8×10^{-2}	0.84	2.4×10^{-3}	0.96	3.9×10^{-1}
Q8BHG2	RIKEN cDNA 0610037L13 gene (0610037L13Rik)	0.96	4.4×10^{-1}	0.86	7.9×10^{-3}	0.93	1.6×10^{-1}
P06151	Lactate dehydrogenase A (Ldha)	0.93	2.1×10^{-2}	0.87	2.8×10^{-3}	0.98	6.1×10^{-1}
P05064	Aldolase A, fructose-bisphosphate (Aldoa)	0.92	6.1×10^{-2}	0.88	7.7×10^{-3}	1.02	1.0×10^{-1}
Q9JKB1	Ubiquitin carboxyl-terminal esterase L3 (ubiquitin thiolesterase) (Uchl3)	0.96	1.1×10^{-1}	0.89	8.6×10^{-3}	0.97	6.9×10^{-1}

Supplementary Table 8: Results of gene ontology analysis for the soleus (Sol) muscle proteins, which were significantly affected by fructo-oligosaccharide (FOS) ingestion. Gene ontology analysis was performed using DAVID Bioinformatics Resources 6.8. The results for the significant higher-abundance of proteins ($p < 0.01$) in Sol muscles of mice exposed to artificial 1-*g* (A1-*g*) with FOS ingestion than those of mice exposed to A1-*g* without FOS ingestion are depicted. The indices in biological process and cellular component with Benjamini values less than 0.05 are shown. The results for the low-abundance proteins ($p < 0.01$) in mice exposed to A1-*g* with FOS ingestion than those in mice exposed to A1-*g* without FOS ingestion did not meet the criterion.

For the high-abundance of proteins in A1-*g* & FOS (+) vs. A1-*g* & FOS (−) groups

**Term**	**No. of proteins**	**Benjamini value**
**Biological process**		
None	None	None
**Cellular component**		
Cytoplasm	17	2.30×10^{-3}
Extracellular exosome	11	2.70×10^{-3}
Myosin filament	3	2.90×10^{-3}
Myelin sheath	4	1.80×10^{-2}

Supplementary Table 9: Responses of soleus (Sol) muscle proteins composing Z-disc to microgravity (μ-*g*) or artificial 1-*g* (A1-*g*) exposure and/or fructo-oligosaccharide (FOS) ingestion. The protein abundance profiles in Sol muscles of the groups exposed to μ-*g* with and without FOS ingestion and the group exposed to A1-*g* with FOS ingestion were individually compared with that of the group exposed to A1-*g* without FOS ingestion. The results for the proteins, which were categorized as the components of Z-disk by DAVID Bioinformatics Resources 6.8 and significantly ($p < 0.01$) affected by μ-*g* exposure and/or FOS ingestion, are indicated. The significant high- and low-abundance of proteins are indicated in orange and blue, respectively.

Accession	Gene name	μ-*g* & FOS (−) /A1-*g* & FOS (−)		μ-*g* & FOS (+) /A1-*g* & FOS (−)		A1-*g* & FOS (+) /A1-*g* & FOS (−)	
		Fold change	*P* value	Fold change	*P* value	Fold change	*P* value
O88990	Actinin alpha 3 (Actn3)	2.66	3.7×10^{-5}	1.82	1.4×10^{-3}	1.08	5.2×10^{-1}
P05064	Aldolase A, fructose-bisphosphate (Aldoa)	1.78	1.5×10^{-4}	1.53	4.8×10^{-3}	0.96	5.1×10^{-1}
Q02357	Ankyrin 1, erythroid (Ank1)	1.10	3.1×10^{-1}	1.37	8.8×10^{-3}	0.92	3.4×10^{-1}
Q9JLV1	BCL2-associated athanogene 3 (Bag3)	1.09	3.1×10^{-1}	1.39	5.8×10^{-4}	1.06	1.6×10^{-1}
Q9R059	Four and a half LIM domains 3 (Fhl3)	1.40	1.1×10^{-2}	1.38	2.3×10^{-3}	1.13	3.7×10^{-1}
P70695	Fructose bisphosphatase 2 (Fbp2)	2.46	1.5×10^{-2}	2.71	2.4×10^{-3}	1.16	3.4×10^{-1}
P14602	Heat shock protein 1 (Hspb1)	1.59	1.3×10^{-3}	1.80	2.7×10^{-3}	1.13	1.1×10^{-1}
Q9ET80	Junctophilin 1 (Jph1)	1.31	1.9×10^{-2}	1.46	2.7×10^{-3}	0.96	6.4×10^{-1}
Q9WUB3	Muscle glycogen phosphorylase (Pygm)	1.64	2.9×10^{-3}	1.39	2.2×10^{-2}	0.89	1.3×10^{-1}
A2AMM0	Muscle-related coiled-coil protein (Murc)	1.37	1.4×10^{-2}	1.44	6.2×10^{-4}	1.02	7.3×10^{-1}

O70209	PDZ and LIM domain 3 (Pdlim3)	1.29	1.8×10^{-3}	1.29	1.4×10^{-2}	1.10	1.6×10^{-1}
P63328	Protein phosphatase 3, catalytic subunit, alpha isoform (Ppp3ca)	2.16	4.7×10^{-4}	2.28	3.6×10^{-3}	0.93	6.3×10^{-1}
Q9JI91	Actinin alpha 2 (Actn2)	0.82	1.1×10^{-3}	0.88	9.6×10^{-4}	1.09	7.5×10^{-2}
P23927	Crystallin, alpha B (Cryab)	0.48	1.1×10^{-3}	0.54	1.7×10^{-5}	1.10	8.7×10^{-2}
P31001	Desmin (Des)	0.52	2.6×10^{-4}	0.51	2.6×10^{-4}	1.07	2.9×10^{-1}
Q9CQM9	Glutaredoxin 3 (Glrx3)	0.82	2.6×10^{-3}	0.94	2.5×10^{-1}	0.91	2.1×10^{-1}
Q9JKS4	LIM domain binding 3 (Ldb3)	0.76	4.5×10^{-3}	0.81	2.0×10^{-2}	1.05	2.5×10^{-1}
Q91Z83	Myosin, heavy polypeptide 7, cardiac muscle, beta (Myh7)	0.77	3.5×10^{-2}	0.83	4.4×10^{-3}	1.38	3.7×10^{-4}
Q9JIF9	Myotilin (Myot)	0.65	8.3×10^{-5}	0.67	6.4×10^{-4}	1.10	1.4×10^{-2}
Q9JJW5	Myozenin 2 (Myoz2)	0.53	7.5×10^{-5}	0.64	1.9×10^{-3}	1.09	1.0×10^{-1}
Q8CI51	PDZ and LIM domain 5 (Pdlim5)	0.78	8.9×10^{-3}	0.84	6.2×10^{-2}	0.98	7.3×10^{-1}
Q8BG95	Protein phosphatase 1, regulatory (inhibitor) subunit 12B (Ppp1r12b)	0.64	7.2×10^{-3}	0.62	3.7×10^{-2}	1.12	6.2×10^{-2}
Q8CC35	Synaptopodin (Synpo)	0.37	1.6×10^{-4}	0.40	5.8×10^{-4}	1.03	6.3×10^{-1}
Q91YE8	Synaptopodin 2 (Synpo2)	0.60	5.7×10^{-4}	0.53	1.5×10^{-3}	1.04	4.0×10^{-1}
Q8BWB1	Synaptopodin 2-like (Synpo2l)	0.61	9.2×10^{-5}	0.56	1.3×10^{-4}	1.14	2.1×10^{-2}

Supplementary Figure Legends

Supplementary Figure 1: Effects of spaceflight on body weight and wet weight of skeletal muscles in mice. The data of mice fed a diet with (n = 3) or without (n = 3) fructo-oligosaccharide (FOS) are integrated in each group and presented as the mean ± standard deviation (SD) (n = 6). Body weight, absolute wet weight of soleus (Sol) and extensor digitorum longus (EDL) muscles, and the wet weight of Sol and EDL muscles relative to body weight are depicted in panels **a**, **b**, and **c**, respectively. Ground control (GC): group of mice raised in the ground model of the Multiple Artificial-gravity Research System (MARS) habitat and transportation cage units; artificial 1-*g* (A1-*g*): group of mice raised under A1-*g* in the International Space Station (ISS); microgravity (μ-*g*): group of mice raised under μ-*g* in the ISS.

Supplementary Figure 2: The effects of microgravity (μ-*g*) or artificial 1-*g* (A1-*g*) exposure on the abundance profiles of proteins in soleus (Sol) and extensor digitorum longus (EDL) muscles of mice, fed a diet with or without fructo-oligosaccharide (FOS). Volcano plots of the proteins identified in Sol and EDL muscles, with the exception of the proteins for which fold changes were “Infinite”, are depicted.

The plots aid visualization of the effects of exposure to μ-*g* or A1-*g* with or without FOS on the abundance profile of muscle proteins in mice.

Supplementary Figure 1

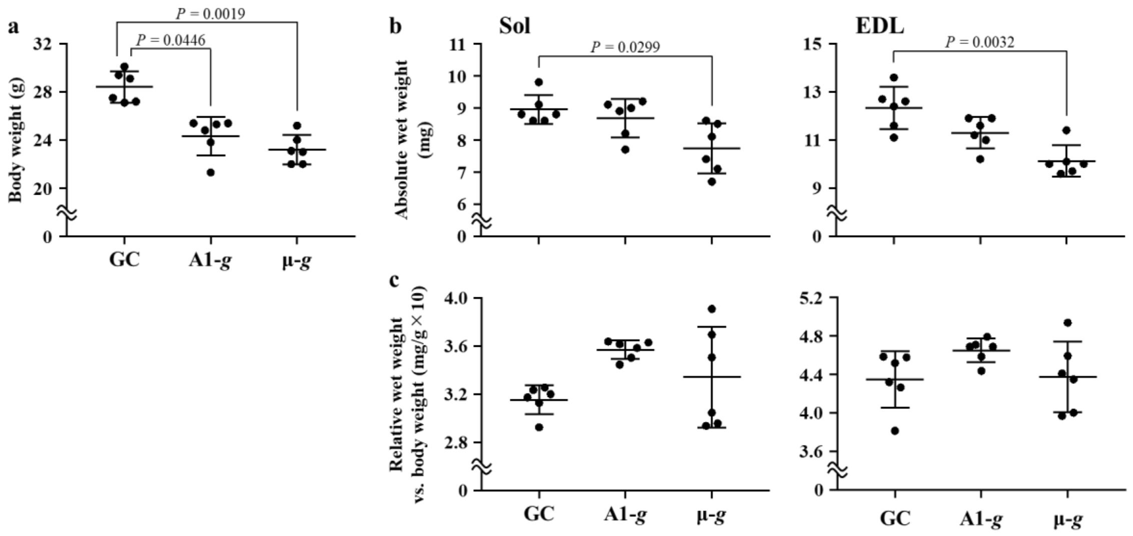

Supplementary Figure 2

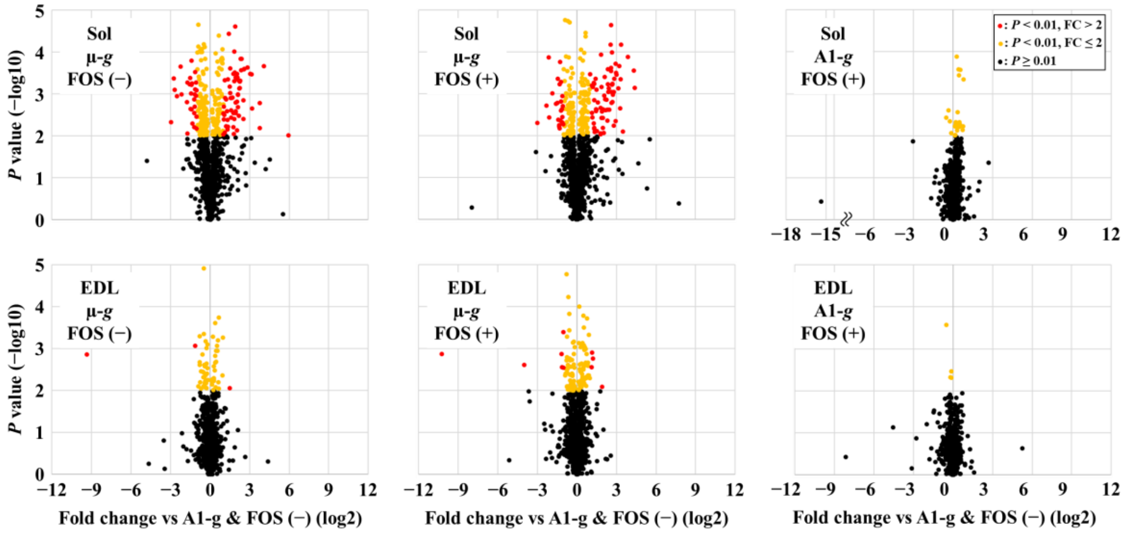

Supplement: Supplementary file 1 — Supplementary information [file 41526_2021_164_MOESM1_ESM.pdf]
