# Supplementary material for: Effects of microgravity exposure and fructo-oligosaccharide ingestion on the proteome of soleus and extensor digitorum longus muscles in developing mice
Source: NPJ Microgravity. 2021 Sep 17;7:34. doi: 10.1038/s41526-021-00164-6 (PMC8448765; doi:10.1038/s41526-021-00164-6)
Supplement: Supplementary file 2 — Reporting Summary [file 41526_2021_164_MOESM2_ESM.pdf]

nature research

Corresponding author(s): Takashi Ohira
Last updated by author(s): Aug 24, 2021

Reporting Summary

Nature Research wishes to improve the reproducibility of the work that we publish. This form provides structure for consistency and transparency in reporting. For further information on Nature Research policies, see our Editorial Policies and the Editorial Policy Checklist.

Statistics

For all statistical analyses, confirm that the following items are present in the figure legend, table legend, main text, or Methods section.

n/a	Confirmed	
☐	☒	The exact sample size (*n*) for each experimental group/condition, given as a discrete number and unit of measurement
☐	☒	A statement on whether measurements were taken from distinct samples or whether the same sample was measured repeatedly
☐	☒	The statistical test(s) used AND whether they are one- or two-sided<br>*Only common tests should be described solely by name; describe more complex techniques in the Methods section.*
☒	☐	A description of all covariates tested
☐	☒	A description of any assumptions or corrections, such as tests of normality and adjustment for multiple comparisons
☐	☒	A full description of the statistical parameters including central tendency (e.g. means) or other basic estimates (e.g. regression coefficient) AND variation (e.g. standard deviation) or associated estimates of uncertainty (e.g. confidence intervals)
☒	☐	For null hypothesis testing, the test statistic (e.g. *F*, *t*, *r*) with confidence intervals, effect sizes, degrees of freedom and *P* value noted<br>*Give P values as exact values whenever suitable.*
☒	☐	For Bayesian analysis, information on the choice of priors and Markov chain Monte Carlo settings
☒	☐	For hierarchical and complex designs, identification of the appropriate level for tests and full reporting of outcomes
☒	☐	Estimates of effect sizes (e.g. Cohen's *d*, Pearson's *r*), indicating how they were calculated

Our web collection on statistics for biologists contains articles on many of the points above.

Software and code

Policy information about availability of computer code

Data collection	Proteomic analysis of mouse skeletal muscle samples was performed using Orbitrap Elite, Hybrid Ion Trap-Orbitrap Mass Spectrometer (Thermo Fisher Scientific Inc.). The mass spectrometer was operated using LTQ Tune Plus (ver.2.7.0.1112 SP2; Thermo Fisher Scientific Inc.). Database searches to identify proteins in the samples were performed using MASCOT (version 2.5.1; Matrix Science, London, UK) against Mus musculus protein sequences (17,034 sequences) in the UniProt Knowledgebase database released November, 2019.
Data analysis	GraphPad Prism version 7.02 software (GraphPad Software, La Jolla, CA, USA); Progenesis QI for proteomics software (version 2.0; Nonlinear Dynamics, Newcastle, UK); Perseus software (http://www.perseus-framework.org); DAVID Bioinformatics Resources 6.8 (https://david.ncifcrf.gov/); Ingenuity Pathway Analysis (version 60467501; Qiagen, Hilden, Germany)

For manuscripts utilizing custom algorithms or software that are central to the research but not yet described in published literature, software must be made available to editors and reviewers. We strongly encourage code deposition in a community repository (e.g. GitHub). See the Nature Research guidelines for submitting code & software for further information.

Data

Policy information about availability of data

All manuscripts must include a data availability statement. This statement should provide the following information, where applicable:

- Accession codes, unique identifiers, or web links for publicly available datasets
- A list of figures that have associated raw data
- A description of any restrictions on data availability

The data that support the findings of this study are available from the corresponding authors upon reasonable request.

Field-specific reporting

Please select the one below that is the best fit for your research. If you are not sure, read the appropriate sections before making your selection.

☒ Life sciences ☐ Behavioural & social sciences ☐ Ecological, evolutionary & environmental sciences

For a reference copy of the document with all sections, see nature.com/documents/nr-reporting-summary-flat.pdf

Life sciences study design

All studies must disclose on these points even when the disclosure is negative.

Sample size	We used the Multiple Artificial-gravity Research System (MARS), which was developed by the Japan Aerospace Exploration Agency (JAXA), for raising mice on the International Space Station (ISS) under microgravity or artificial 1-g. A maximum of only 12 mice can be raised using the MARS. The main goal of the second mission using the MARS, which was organized by JAXA, was to evaluate the effects of microgravity exposure on the proteome of mouse skeletal muscles and verify the efficacy of fructo-oligosaccharide ingestion (FOS) as a countermeasure for the spaceflight-related deterioration of muscle function. Therefore, 12 mice were randomly divided into 2 groups and individually raised under microgravity or artificial 1-g, and 3 mice in each group were fed a diet containing 5% FOS. We could analyze only the skeletal muscle samples from these mice (n = 3/group). Additionally, it is not feasible to collect additional skeletal muscle samples from space-flown mice. This is a study limitation.
Data exclusions	No data were excluded from the analyses.
Replication	We individually analyzed skeletal muscle protein extracts one time each. We extracted skeletal muscle proteins by a two-step solubilization method using 50 mM Tris-HCl buffer (pH 7.5) containing protease inhibitor, and a lysis buffer containing 8 M urea, 50 mM NH4HCO3, 4% sodium deoxycholate, and protease inhibitor. Then, protein abundance profiles in both fractions, i.e., supernatant-1 and supernatant-2, were individually determined using Orbitrap Elite, Hybrid Ion Trap-Orbitrap Mass Spectrometer (Thermo Fisher Scientific Inc.). In our previous study (Ohira et al., J Ptoteomics, 2020), proteomic analysis of three different sections obtained from a mouse gastrocnemius muscle revealed that proteomic analysis using the two-step protein solubilization method had low variation and high reproducibility in protein detection and peptide quantification. Therefore, we did not replicate the analysis in this study. However, we individually analyzed protein extracts from all skeletal muscle samples to increase the reliability of our results.
Randomization	Twelve mice were randomly divided into 2 groups and individually raised under microgravity or artificial 1-g on the ISS, and 3 mice, which were randomly selected, in each group were fed a diet containing 5% FOS.
Blinding	Data collection and analysis were led by a different person. The investigators were blinded to group allocation during data collection. However, the investigators needed to know group allocation during data analysis to evaluate the effects of microgravity exposure and FOS ingestion on the proteome of mouse skeletal muscles (n = 3/group). The obtained results were checked and interpleted by several investigators to secure the objectivity.

Reporting for specific materials, systems and methods

We require information from authors about some types of materials, experimental systems and methods used in many studies. Here, indicate whether each material, system or method listed is relevant to your study. If you are not sure if a list item applies to your research, read the appropriate section before selecting a response.

Materials & experimental systems

n/a	Involved in the study	
☒	☐	Antibodies
☒	☐	Eukaryotic cell lines
☒	☐	Palaeontology and archaeology
☐	☒	Animals and other organisms
☒	☐	Human research participants
☒	☐	Clinical data
☒	☐	Dual use research of concern

Methods

n/a	Involved in the study	
☒	☐	ChIP-seq
☒	☐	Flow cytometry
☒	☐	MRI-based neuroimaging

Animals and other organisms

Policy information about studies involving animals; ARRIVE guidelines recommended for reporting animal research

Laboratory animals	We purchased male C57BL/6J mice, aged 5 weeks, from Jackson Laboratory (Bar Harbor, ME, USA) and used in this study.
Wild animals	*Provide details on animals observed in or captured in the field; report species, sex and age where possible. Describe how animals were caught and transported and what happened to captive animals after the study (if killed, explain why and describe method; if released, say where and when) OR state that the study did not involve wild animals.*

Field-collected samples	*For laboratory work with field-collected samples, describe all relevant parameters such as housing, maintenance, temperature, photoperiod and end-of-experiment protocol OR state that the study did not involve samples collected from the field.*
Ethics oversight	*Identify the organization(s) that approved or provided guidance on the study protocol, OR state that no ethical approval or guidance was required and explain why not.*

Note that full information on the approval of the study protocol must also be provided in the manuscript.
